# Association of a Schizophrenia-Risk Nonsynonymous Variant With Putamen Volume in Adolescents: A Voxelwise and Genome-Wide Association Study

**DOI:** 10.1001/jamapsychiatry.2018.4126

**Published:** 2019-01-16

**Authors:** Qiang Luo, Qiang Chen, Wenjia Wang, Sylvane Desrivières, Erin Burke Quinlan, Tianye Jia, Christine Macare, Gabriel H. Robert, Jing Cui, Mickaël Guedj, Lena Palaniyappan, Ferath Kherif, Tobias Banaschewski, Arun L. W. Bokde, Christian Büchel, Herta Flor, Vincent Frouin, Hugh Garavan, Penny Gowland, Andreas Heinz, Bernd Ittermann, Jean-Luc Martinot, Eric Artiges, Marie-Laure Paillère-Martinot, Frauke Nees, Dimitri Papadopoulos Orfanos, Luise Poustka, Juliane H. Fröhner, Michael N. Smolka, Henrik Walter, Robert Whelan, Joseph H. Callicott, Venkata S. Mattay, Zdenka Pausova, Jean-François Dartigues, Christophe Tzourio, Fabrice Crivello, Karen F. Berman, Fei Li, Tomáš Paus, Daniel R. Weinberger, Robin M. Murray, Gunter Schumann, Jianfeng Feng

**Affiliations:** 1 Institute of Science and Technology for Brain-Inspired Intelligence, Fudan University, Shanghai, China; 2 Ministry of Education-Key Laboratory of Computational Neuroscience and Brain-Inspired Intelligence, Fudan University, Shanghai, China; 3 School of Life Sciences and State Key Laboratory of Genetic Engineering, Fudan University, Shanghai, China; 4 Centre for Population Neuroscience and Precision Medicine, Institute of Psychiatry, Psychology and Neuroscience, King’s College London, Social Genetic and Developmental Psychiatry Centre, London, England; 5 Lieber Institute for Brain Development, Johns Hopkins Medical Campus, Baltimore, Maryland; 6 Pharnext, Issy-les-Moulineaux, Ile de France, France; 7 Institut National de la Santé et de la Recherche Médicale Unit 897, University of Bordeaux, Bordeaux, Aquitaine, France; 8 EA 4712 “Behavior and Basal Ganglia,” Rennes University 1, Rennes, France; 9 Laboratory for Research in Neuroimaging, Department of Clinical Neurosciences, Centre Hospitalier Universitaire Vaudois, University of Lausanne, Lausanne, Switzerland; 10 Departments of Psychiatry and Medical Biophysics, Robarts Research Institute, University of Western Ontario, London, Ontario, Canada; 11 Department of Child and Adolescent Psychiatry and Psychotherapy, Central Institute of Mental Health, Medical Faculty Mannheim, Heidelberg University, Square J5, Mannheim, Germany; 12 Discipline of Psychiatry, School of Medicine and Trinity College Institute of Neuroscience, Trinity College Dublin, Dublin, Ireland; 13 University Medical Centre Hamburg-Eppendorf, Hamburg, Germany; 14 Department of Cognitive and Clinical Neuroscience, Central Institute of Mental Health, Medical Faculty Mannheim, Heidelberg University, Mannheim, Germany; 15 Department of Psychology, School of Social Sciences, University of Mannheim, Mannheim, Germany; 16 NeuroSpin, Commissariat à L'énergie Atomique, Université Paris-Saclay, Gif-sur-Yvette, France; 17 Departments of Psychiatry and Psychology, University of Vermont, Burlington,; 18 Sir Peter Mansfield Imaging Centre School of Physics and Astronomy, University of Nottingham, University Park, Nottingham, England; 19 Department of Psychiatry and Psychotherapy, Campus Charité Mitte, Charité, Universitätsmedizin Berlin, Germany; 20 Physikalisch-Technische Bundesanstalt Braunschweig and Berlin, Berlin, Germany; 21 Institut National de la Santé et de la Recherche Médicale Unit 1000, Neuroimaging and Psychiatry, University Paris Sud–Paris Saclay, University Paris Descartes, Paris, France; 22 Service Hospitalier Frédéric Joliot, Orsay, France; 23 Maison de Solenn, Paris, France; 24 GH Nord Essonne Psychiatry Department, Orsay, France; 25 Assistance Publique–Hôpitaux de Paris, Department of Child and Adolescent Psychiatry, Pitié-Salpêtrière Hospital, Paris, France; 26 Department of Child and Adolescent Psychiatry and Psychotherapy, University Medical Centre Göttingen, Göttingen, Germany; 27 Clinic for Child and Adolescent Psychiatry, Medical University of Vienna, Währinger Gürtel, Vienna, Austria; 28 Department of Psychiatry and Neuroimaging Center, Technische Universität Dresden, Dresden, Germany; 29 School of Psychology and Global Brain Health Institute, Trinity College Dublin, Dublin, Ireland; 30 Clinical and Translational Neuroscience Branch, National Institute of Mental Health, National Institutes of Health, Bethesda, Maryland; 31 Departments of Neurology, Johns Hopkins University School of Medicine, Baltimore, Maryland; 32 Departments of Radiology, Johns Hopkins University School of Medicine, Baltimore, Maryland; 33 The Hospital for Sick Children, University of Toronto, Toronto, Ontario, Canada; 34 Institut National de la Santé et de la Recherche Médicale Unit 1219, Université de Bordeaux, Bordeaux, France; 35 University de Bordeaux, Institut des Maladies Neurodégénératives, Bordeaux, France; 36 Centre National de la Recherche Scientifique, Institut des Maladies Neurodégénératives, Bordeaux, France; 37 Commissariat à L'énergie Atomiquecea, Institut des Maladies Neurodégénératives-Equipe 5, Bordeaux, France; 38 Developmental and Behavioral Pediatric Department and Child Primary Care Department, MOE-Shanghai Key Lab for Children's Environmental Health, Xinhua Hospital Affiliated To Shang Jiaotong University School of Medicine, Shanghai, China; 39 Bloorview Research Institute, Holland Bloorview Kids Rehabilitation Hospital, Toronto, Ontario, Canada; 40 Departments of Psychology and Psychiatry, University of Toronto, Toronto, Ontario, Canada; 41 McKusick Nathans Institute of Genetic Medicine, Johns Hopkins School of Medicine, Baltimore, Maryland; 42 Department of Psychiatry and Behavioral Sciences, Johns Hopkins University School of Medicine, Baltimore, Maryland; 43 Department of Neuroscience, Johns Hopkins School of Medicine, Baltimore, Maryland; 44 Department of Computer Science, University of Warwick, Coventry, England; 45 Collaborative Innovation Center for Brain Science, Fudan University, Shanghai, China; 46 Shanghai Center for Mathematical Sciences, Shanghai, China

## Abstract

**Importance:**

Deviation from normal adolescent brain development precedes manifestations of many major psychiatric symptoms. Such altered developmental trajectories in adolescents may be linked to genetic risk for psychopathology.

**Objective:**

To identify genetic variants associated with adolescent brain structure and explore psychopathologic relevance of such associations.

**Design, Setting, and Participants:**

Voxelwise genome-wide association study in a cohort of healthy adolescents aged 14 years and validation of the findings using 4 independent samples across the life span with allele-specific expression analysis of top hits. Group comparison of the identified gene-brain association among patients with schizophrenia, unaffected siblings, and healthy control individuals. This was a population-based, multicenter study combined with a clinical sample that included participants from the IMAGEN cohort, Saguenay Youth Study, Three-City Study, and Lieber Institute for Brain Development sample cohorts and UK biobank who were assessed for both brain imaging and genetic sequencing. Clinical samples included patients with schizophrenia and unaffected siblings of patients from the Lieber Institute for Brain Development study. Data were analyzed between October 2015 and April 2018.

**Main Outcomes and Measures:**

Gray matter volume was assessed by neuroimaging and genetic variants were genotyped by Illumina BeadChip.

**Results:**

The discovery sample included 1721 adolescents (873 girls [50.7%]), with a mean (SD) age of 14.44 (0.41) years. The replication samples consisted of 8690 healthy adults (4497 women [51.8%]) from 4 independent studies across the life span. A nonsynonymous genetic variant (minor T allele of rs13107325 in *SLC39A8*, a gene implicated in schizophrenia) was associated with greater gray matter volume of the putamen (variance explained of 4.21% in the left hemisphere; 8.66; 95% CI, 6.59-10.81; *P* = 5.35 × 10^−18^; and 4.44% in the right hemisphere; *t* = 8.90; 95% CI, 6.75-11.19; *P* = 6.80 × 10^−19^) and also with a lower gene expression of *SLC39A8* specifically in the putamen (t_127_ = −3.87; *P* = 1.70 × 10^−4^). The identified association was validated in samples across the life span but was significantly weakened in both patients with schizophrenia (*z* = −3.05; *P* = .002; n = 157) and unaffected siblings (*z* = −2.08; *P* = .04; n = 149).

**Conclusions and Relevance:**

Our results show that a missense mutation in gene *SLC39A8* is associated with larger gray matter volume in the putamen and that this association is significantly weakened in schizophrenia. These results may suggest a role for aberrant ion transport in the etiology of psychosis and provide a target for preemptive developmental interventions aimed at restoring the functional effect of this mutation.

## Key Points

**Question:**

Is there any genetic variant associated with adolescent brain development that can inform psychopathology of schizophrenia?

**Findings:**

In this imaging genetics study of brain structure, a significant association between a missense mutation in *SLC39A8* (a gene previously associated with schizophrenia) and gray matter volume in putamen was discovered and replicated using 10 411 healthy participants from 5 independent studies. Compared with healthy control individuals, such association was significantly weakened in both patients with schizophrenia and unaffected siblings.

**Meaning:**

Common genetic variant indicates an involvement of neuronal ion transport in both pathophysiology of schizophrenia and structural development of putamen.

## Teaser

This imaging genetics study examines genetic variants associated with adolescent brain structure and explores the psychopathologic relevance of such associations in the development of schizophrenia.

## Introduction

The adolescent brain undergoes substantial structural change, and deviations from the normal trajectory of brain development are thought to underlie many psychiatric symptoms.^1^ Growth patterns of adolescent brain development have been identified using longitudinal neuroimaging studies: decrease (eg, cortical regions, caudate, and putamen), increase (eg, hippocampus), and inverted U-shaped (eg, amygdala and thalamus).^2,3,4,5^ Twin studies have demonstrated regionally specific changes in heritability during different phases of brain development,^6^ and significant age-by-heritability interactions have been reported for gray matter volumes (GMV) in cortical and subcortical structures.^7^ Common genetic associations with both adolescent brain structures and risks for psychiatric disorders remain to be uncovered.

Large-scale meta-analysis of genome-wide association study (GWAS) is the state-of-the-art approach to detect novel genetic variants associated with brain structure. However, often these studies are carried out in samples from heterogeneous age groups to maximize the overall sample size,^8^ and large-scale GWAS on adolescent brain is not available yet. Thus, much less is known about genetic factors to provide us with information about normal trajectories of brain development, and deviations from normal trajectories have been implicated in the pathophysiology of mental disorders.^9,10,11^ To increase the statistical power to detect genetic associations in the developing adolescent brain, it is important to investigate a sample with a narrow age range.^10^ This has already been demonstrated in a 2014 twin study,^12^ in which the heritability estimated from 89 twin pairs at the same age resembled estimates given by large meta-analysis, with more than 1250 twin pairs from different age groups.^13^ Additional limitations in detecting genetic associations might have been caused by using atlas-based brain segmentation because brain regions such defined can be genetically heterogeneous,^14^ thus potentially resulting in false-negative observations. To address these limitations, we investigated a cohort of more than 2000 healthy adolescents aged 14 years (IMAGEN^15^) and combined voxelwise brain imaging with genome-wide association study (vGWAS^16^).

Genetic associations on brain structures can emerge in a particular developmental period or can present across the life span.^6,7^ Thus, genetic factors might cause pervasive neuroanatomical aberrations that are linked to psychopathology during a defined developmental period or across the life span.^9,10,11^ To validate our findings and extend them to a wider age range, we used 4 additional cohorts of healthy participants to characterize patterns of the identified associations across the life span including the Saguenay Youth Study (SYS^17^), Lieber Institute for Brain Development sample (LIBD^18^), UK Biobank (UKB^19^), and Three-City Study (3C^20^). For the identified genetic variants, we tested their cisregulations on the expressions of nearby genes in brain tissues. To test whether genetic associations of adolescent brain are disrupted by psychopathology, we compared the identified associations among patients with psychiatric disorder, unaffected siblings, and healthy control individuals in clinical sample.

## Method

### Participants

#### Discovery Sample and Samples Across the Life Span

The IMAGEN study,^15^ a population-based longitudinal imaging genetics cohort, recruited 2087 healthy adolescents aged 14 years, of which 1721 entered the vGWAS (eMethods 1 and 2 in the Supplement). We also investigated 971 healthy participants from the adolescent SYS sample,^17^ 272 healthy participants from the clinical LIBD sample,^18^ 6932 participants from the population-based UKB cohort,^19^ and 515 healthy elderly participants from the 3C sample,^21^ a population-based cohort study (eMethods 3-6 in the Supplement).

#### Clinical Sample

In the LIBD study of schizophrenia,^18^ we investigated 157 treated patients with chronic schizophrenia and 149 unaffected siblings of patients (eMethods 4 in the Supplement). The IMAGEN project had obtained ethical approval by the local ethics committees, including King’s College London, University of Nottingham, Trinity College Dublin, University of Heidelberg, Technische Universität Dresden, Commissariat à l'Energie Atomique et aux Energies Alternatives, and University Medical Center, University of Hamburg, Hamburg, Germany. For SYS, the institutional review boards of all participating institutions approved all studies reported herein. The participants of the LIBD study were recruited as part the Clinical Brain Disorders Branch Sibling Study of schizophrenia at the National Institute of Mental Health (Daniel R. Weinberger, principal investigator). The study was approved by the institutional review board of the Intramural Program of the National Institute of Mental Health. The 3C study was approved by the Ethics Committee of the Hôpital de Bicêtre. All adult participants provided written informed consent after information on the research procedures by each cohort study. For adolescent participants in IMAGEN and SYS, all participants’ parents provided written informed consent after information on the research procedures and adolescents provided their assent after written information.

### Measures

#### Genome-Wide Genotype Data

The IMAGEN blood samples were genotyped using either Illumina Human610-Quad Beadchip or Illumina Human660-Quad Beadchip. After quality control, 466 114 single-nucleotide polymorphisms (SNPs) entered the following analysis. Details of the genotyping and quality control are available in a publication^22^ and in eMethods 1 in the Supplement.

#### Structural Image Data

Structural magnetic resonance imaging (MRI) was performed on 3-T scanners from 3 manufacturers (Siemens: 5 sites; Philips: 2 sites; and General Electric: 2 sites) following the Alzheimer’s Disease Neuroimaging Initiative protocol modified for the IMAGEN study. All data were preprocessed in Statistical Parametric Mapping, version 8 using the Voxel-Based Morphometry, version 8 toolbox, including segmentation, normalization, modulation, and smoothing (eMethods 2 in the Supplement).

#### Brain Expression Quantitative Trait Loci Database

In the UK Brain Expression Consortium (UKBEC^23^) database, gene expression data are available for 10 brain regions from 134 neuropathologically free participants. For any vGWAS-identified mutation on a gene, we first tested whether this SNP was associated with expression of this gene. Second, we went on to test whether such an association was tissue specific and whether this SNP also had cisregulations on expressions of nearby (±1 Mb) genes. For this extended exploration, we corrected for multiple comparisons between the number of nearby genes and the number of brain areas (eMethods 7 in the Supplement).

### Statistical Analysis

#### Voxelwise and Genome-Wide Association Study

On the discovery sample, we performed a GWAS on GMV of each voxel in the brain (ie, 438 145 voxels labeled as per the Automatically Anatomical Labeling template^24^). A significant association was identified if a cluster had more than 217 (approximately 4/3 × π × [3.3970 × 1.645]^3^/1.5^3^ voxels falling into the 90% confidence interval of the smoothing kernel) voxels with 2-sided *P* values surviving a Bonferroni correction (*P* < 2.4483 × 10^−13^, calculated by 0.05/438 145/466 114; eMethods 8 in the Supplement). Regions of interest were then established from the identified clusters, and GMV of each region of interest was calculated by adding the volumes of all voxels within this region. Replications were mainly conducted for the significant clusters using each replication sample (eMethods 9 in the Supplement for meta-analysis). We established the 95% confidence interval of the statistics by 3000 bootstraps.

#### Summary-Databased Mendelian Randomization

For the identified brain structure, we conducted summary-databased Mendelian randomization (SMR) analysis by a web-based application (MR-Base^25^; eMethods 10 in the Supplement). Using Psychiatric Genomics Consortium 2014 GWAS results for schizophrenia^26^ as the outcome, we tested whether the association between the identified brain structure and schizophrenia was significant and free of nongenetic confounders.^27^ A significant SMR result may suggest an association between the exposure (brain volume) and the outcome (schizophrenia) using the exposure-associated genetic variant as an instrument because the random nature of genetic variation mimics the design of randomized clinical trials.^25^ Although significant SMR results require further biological validation, nonsignificant results at least indicate a lack of association.^28^

#### Comparison Among Patients, Unaffected Siblings, and Healthy Control Individuals

We first conducted power analysis to test whether we had enough sample size to detect the previously identified genetic associations in our clinical sample (eMethods 11 in the Supplement). To compare the identified association in patients with schizophrenia or unaffected siblings with that in healthy control individuals, we estimated its effect size using correlation coefficient. Partial correlations between GMV of the regions of interest and SNPs were estimated controlling for age, age × age, sex, IQ, total intracranial volume, and ratio of gray and white matter volume over total intracranial volume. Between independent samples, we compared effects sizes (ie, partial correlation coefficient) after transforming them into *z* statistics. The 95% 1-sided upper bound was established by 3000 bootstraps for the difference between 2 partial correlations in patients and their paired unaffected siblings, respectively.

## Results

### Demographics

In the discovery sample of 1721 healthy adolescents (of whom 873 were girls [50.7%]), the participants were a mean (SD) age of 14.44 (0.41) years, while the replication samples of 8690 healthy participants (of whom 4497 were girls [51.8%]) had a larger age range between 12 and 92 years. The clinical sample used in this study had 157 patients with schizophrenia (of whom 35 were female [22.2%], with a mean [SD] age of 34.82 [9.91] years) and 149 unaffected siblings of patients (of whom 85 were female [57.1%], with a mean [SD] age of 36.60 [9.44] years). Further demographics and clinical features are listed in eTable 1 in the Supplement.

### Association of Schizophrenia Risk SNP rs13107325 With Putamen Volume

**Figure 1.**
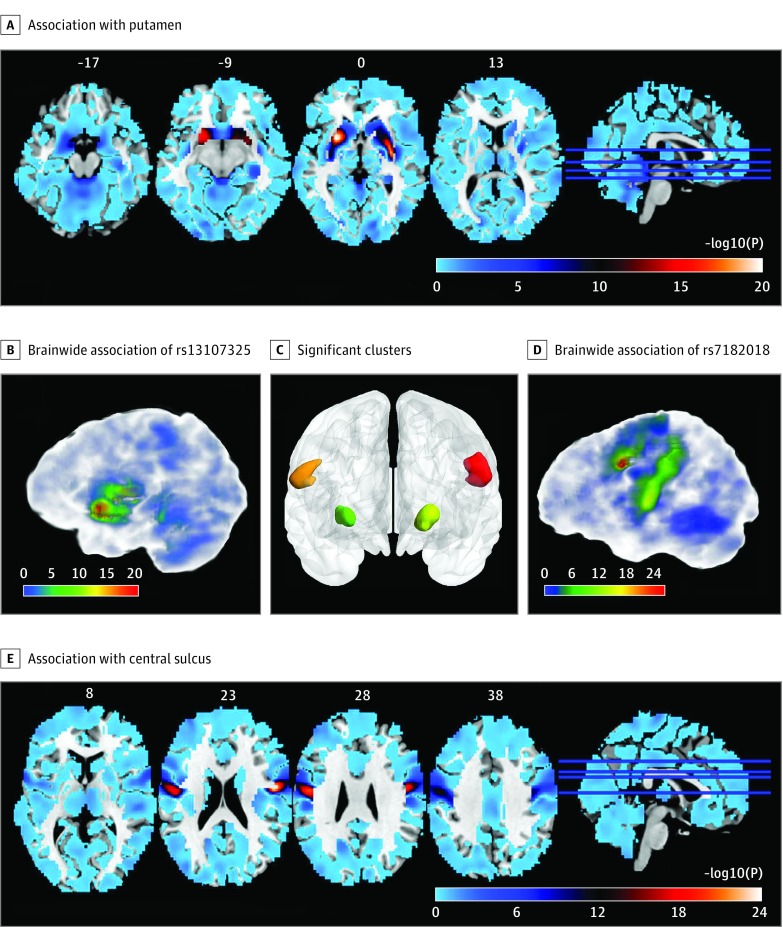
Significant Associations Identified by Voxelwise and Genome-Wide Association Study The significance level of the association (-log_10_ *P*) between the gray matter volume of each voxel of the brain and the SNPs rs13107325 (A and C) or rs7182018 (D and E). Red represents stronger association, while blue represents weaker association. B, Four clusters of voxels survived the Bonferroni correction (*P* < 2.45 × 10^−13^, calculated by 0.05 / 466 114 [number of SNPs] / 438 145 [number of voxels]). Two clusters around the left and right central sulcus are marked in red and orange, respectively. Two clusters in the left and right putamen are marked by yellow and green, respectively.

Applying voxelwise and GWAS (vGWAS) to the discovery sample, we found that the minor T allele (a missense mutation in gene *SLC39A8*) of SNP rs13107325 was associated with larger volumes in bilateral putamen (left hemisphere: *t*_1705_ = 8.66; *P* = 5.35 × 10^−18^; variance explained [VE] = 4.21%; right hemisphere: *t*_1705_ = 8.90; *P* = 6.80 × 10^−19^; VE = 4.44% right hemisphere), and these clusters were asymmetric between left and right hemispheres (Figure 1A-C). In addition, we found an association of the minor G allele of SNP rs7182018 (an intron variant on lncRNA RP11-624L4.1) with greater GMV of 2 clusters in bilateral central sulcus (left hemisphere: *t*_1705_ = 9.86; *P* = 1.25 × 10^−22^; VE = 5.39%; right hemisphere: *t*_1705_ = 9.96; *P* = 4.54 × 10^−23^; VE = 5.50%; Figure 1D and E; Table; eTables 2-4 in the Supplement; eFigure 1 for Manhattan plots and QQ plots in the Supplement; eFigure 2 for distributions and bootstraps in the Supplement).

**Table.** Associations of a Schizophrenia-Risk SNP rs13107325 With the Gray Matter Volumes of 2 Putamen Clusters in Multiple Cohorts^a^

Sample and Cluster	Volume, mean (SD), mL	*t* (95% CI)	*P* Value	Variance Explained, %
IMAGEN^b^				
Left PUT	1.93 (0.35)	8.66 (6.59 to 10.81)	5.35 × 10^−18^	4.21
Right PUT	0.75 (0.09)	8.90 (6.75 to 11.19)	6.80 × 10^−19^	4.44
SYS^c^				
Left PUT	1.60 (0.22)	3.70 (1.85 to 5.60)	1.16 × 10^−4^	1.40
Right PUT	0.81 (0.11)	−1.73 (−3.54 to −0.04)	.08^d^	0.31
LIBD HC^e^				
Left PUT	1.59 (0.22)	4.93 (2.86 to 7.11)	7.22 × 10^−7^	8.38
Right PUT	0.65 (0.06)	5.33 (3.29 to 7.48)	1.05 × 10^−7^	9.65
UKB^f^				
Left PUT	1.37 (0.28)	4.80 (2.97 to 6.72)	8.16 × 10^−7^	0.33
Right PUT	0.53 (0.09)	6.46 (4.48 to 8.41)	5.44 × 10^−11^	0.60
3C^g^				
Left PUT	1.11 (0.14)	2.34 (0.62 to 4.45)	.01	1.07
Right PUT	0.48 (0.06)	2.28 (0.45 to 4.31)	.01	1.02
				
LIBD SZ^h^				
Left PUT	1.57 (0.28)	2.01 (0.60 to 3.55)	.02	2.00
Right PUT	0.65 (0.09)	0.17 (−1.46 to 1.78)	.43	0.02
LIBD SB^i^				
Left PUT	1.53 (0.21)	2.27 (0.23 to 4.09)	.01	3.47
Right PUT	0.63 (0.06)	1.30 (−0.93 to 3.11)	.10	1.16

Abbreviations: 3C, Three-City Study; HC, healthy control individuals; LIBD; Lieber Institute for Brain Development; PUT, putamen; SYS, Saguenay Youth Study; SZ, patients with schizophrenia; SB, unaffected siblings of patients; SNP, single-nucleotide polymorphism; UKB, UK biobank.

^a^ Validations of positive associations in different age groups. *P* values were given by 1-tailed test. The associations were estimated for the volumes of the significant clusters identified by our voxelwise genome-wide association study. The volume of a cluster was calculated by adding up the volume of each voxel within that cluster.

^b^ n = 1721; Mean age, 14 years.

^c^ n = 971; Mean age, 15 years.

^d^ Two-tailed *P *value test because the association went to an opposite direction compared with the hypothesis.

^e^ n = 272; Mean age, 32 years.

^f^ n = 6932; Mean age, 62 years.

^g^ n = 515; Mean age, 77 years.

^h^ n = 157; Mean age, 35 years.

^i^ n = 149; Mean age, 37 years.

rs13107325 has been associated with schizophrenia in a 2014 Psychiatric Genomics Consortium (phase 2) GWAS.^26^ The SMR using Psychiatric Genomics Consortium (phase 2) results as outcome identified the associations between GMVs of the putamen clusters and schizophrenia (left putamen cluster: b = 0.9388; SE = 0.1329; *P* = 1.61 × 10^−12^; right putamen cluster: b = 3.444; SE = 0.4875; *P* = 1.607 × 10^−12^; eFigure 3 in the Supplement). Considering that the SMR analysis identified no association between the central sulcus and schizophrenia using any SNP within the neighboring region (±1 Mb) of rs7182018 as an instrumental variable (eFigure 4 in the Supplement), we concluded that rs7182018 is not associated with schizophrenia. Analyses on rs7182018 are found in eTables 2-12 and eFigures 5-13 in the Supplement.

### Independent Replications Across the Life Span

In the SYS sample of 971 healthy adolescents with a mean (SD) age of 15.03 (1.84) years, we replicated the positive association of SNP rs13107325 in the left putamen (*t*_964_ = 3.70; *P* = 1.16 × 10^−4^) but found no such association in the right putamen (*t*_964_ = −1.73; *P* = .08). The right putamen cluster was affected by a greater variation of the insula in the SYS sample because a part of the insula was mapped into this cluster (eFigure 14 in the Supplement).

Using the UKB sample (mean [SD] age, 62.64 [7.41] years; n = 6932), we replicated the positive associations of rs13107325 with GMV of the putamen clusters (left hemisphere: *t*_6885_ = 4.80; *P* = 8.16 × 10^-^**^7^**; VE = 0.33%; right hemisphere: *t*_6885_ = 4.80; *P* = 8.16 × 10^-^**^7^**; VE = 0.60%). Given the large sample size of this cohort, we further confirmed the significance of the identified clusters using a SNP to whole-brain approach with 10 000 permutations at a cluster level (eTable 11 in the Supplement). In another 2 independent samples with mean (SD) ages of 31.92 (9.50) years (LIBD sample, n = 272) and 77.48 (5.12) years (3C sample, n = 515), we again confirmed the identified positive associations (LIBD sample, left putamen: *t*_264_ = 4.93; *P* = 7.22 × 10^−7^; VE = 8.38%; right putamen: *t*_264_ = 5.33; *P* = 1.05 × 10^−7^; VE = 9.65% ; 3C sample, left putamen: *t*_507_ = 2.34; *P* = .01; VE = 1.07%; right putamen: *t*_507_ = 2.28; *P* = .01; VE = 1.02%; Table; eTables 9 and 10 in the Supplement; and eFigures 7-12 and 15-17 in the Supplement).

### Association of rs13107325 With Lower Expression Level of *SLC39A8* in Putamen

**Figure 2.**
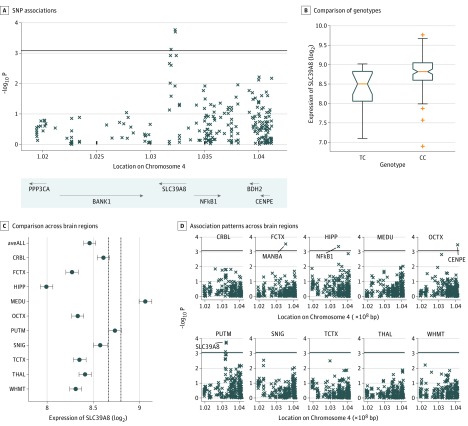
Gene Expression of SLC39A8 at Putamen and Gray Matter Volume at Putamen Shared Common Genetic Controls A, Significance level of associations between single-nucleotide polymorphism (SNP) rs13107325 and gene expression levels of nearby genes of SLC39A8 (the probes used by Affymetrix were organized according to locations of their starting base at chromosome 4). B, Comparison between gene expression levels of SLC39A8 at putamen with different genotypes at SNP rs13107325. C, Comparison on gene expression levels (mean value and 95% confidence interval) of SLC39A8 across 10 brain regions, including inferior olivary nucleus (MEDU; subdissected from the medulla), putamen (PUTM; at the level of the anterior commissure), substantia nigra (SNIG), cerebellar cortex (CRBL), thalamus (THAL; at the level of the lateral geniculate nucleus), temporal cortex (TCTX), intralobular white matter (WHMT), occipital cortex (OCTX), frontal cortex (FCTX), and hippocampus (HIPP). D, Association patterns between SNP rs13107325 and gene expressions in 10 brain regions. Genes with significant associations (*P* < .0008, calculated by 0.05/10/6 by Bonferroni correction) were labeled with gene names. bp Indicates base pairs.

Using the expression quantitative trait loci (eQTL) database from the UKBEC (n = 134, with 112 CC genotypes, 22 CT genotypes, and 0 TT at the SNP rs13107325), we found that the carriers of the risk allele (T) at rs13107325 showed lower expression of *SLC39A8* (*t*_127_ =  −3.87; 95% CI, −6.51 to −1.73; *P* = .0002) in the putamen (Figure 2A and B). Furthermore, we found that despite brainwide expression of *SLC39A8* (Figure 2C), this eQTL association was specific for the putamen and was not detected in any of the other brain regions (*P* < .0008, Bonferroni correction for 10 types of brain tissues and 6 neighboring genes) (Figure 2D). In addition to gene *SLC39A8* (eTables 13 and 14 in the Supplement), we also found associations of rs13107325 with lower gene expressions of *NF-κB1* in the hippocampus (*t*_120_ =  −3.62; 95% CI, −6.31 to −1.28; *P* = .0004), *MANBA* in the frontal cortex (*t*_125_ = −3.73; 95% CI, −5.93 to −1.84; *P* = .0003), and higher expression of *CENPE* in the occipital cortex (*t*_127_ = 3.69; 95% CI 1.72 to 6.10; *P* = .0003).

### Gene-Brain Association Weakened by Genetic Risk for Schizophrenia

Despite inconsistent structural neuroimaging results of the putamen in schizophrenia (no difference,^29,30^ reduction,^31^ or enlargement^32,33,34,35,36^ of structure have been reported), this structure has long been associated with both elevated dopamine synthesis capacity^37,38^ and frontostriatal dysconnectivity^39^ in schizophrenia and is key to the effects of antipsychotic treatment^40,41,42,43^ by various methodologic approaches.^38,39,44,45^ To reduce the confounding effects, we used unaffected siblings (carrying a higher genetic risk for schizophrenia^46^ but free of the clinical phenotype and treatment effects^18^) of patients with schizophrenia to further validate the involvement of the rs13107325-putamen association in schizophrenia. We hypothesized that the rs13107325-putamen association was significantly weakened in both patients and unaffected siblings compared with healthy control individuals. Given a large effect size (*r* = 0.3117; n = 272) in the healthy control individuals, power analysis (eMethods 11 in the Supplement) estimated a sample size of 102 for 95% power assuming a 5% significance level and a 1-sided test. Therefore, we had enough patients (n = 157) and unaffected siblings (n = 149) in the LIBD study to detect such an association. We found that the rs13107325-putamen association in the right hemisphere became insignificant in both patients and unaffected siblings (Table). This disrupting effect might be specific because the rs7182018-CEN association remained significant in all 3 groups (eTable 5 in the Supplement). Compared with healthy control individuals, patients had a significantly weakened rs13107325-putamen association (*z* = −3.05; *P* = .002). Next, we confirmed that such association was weaker in the unaffected siblings compared with the healthy control individuals (*z* = −2.08; *P* = .04). In patient-sibling pairs (n = 49), we found that the SNP-volume association was weaker in patients compared with unaffected siblings (*r*_patient_-*r*_sibling_ = −0.25; 95% upper 1-sided bound; −0.0143; *P* = .04).

## Discussion

In this vGWAS, we discovered an rs13107325-putamen association in adolescent brains and confirmed this association across the life span. Mendelian randomization analysis demonstrated a significant association between putamen volume and schizophrenia free of nongenetic confounders. Unaffected siblings of patients showed a significant weakening of the rs13107325-putamen association that may be owing to the genetic risk for schizophrenia. Together, these findings provide a new and testable hypothesis of an interaction between the pathology of schizophrenia and the mechanism determining the putamen volume.

Single-nucleotide polymorphism rs13107325 (located in an exon of *SLC39A8*, chromosome 4) encodes a solute carrier transporter *ZIP8* expressed in the plasma membrane and mitochondria. *SLC39A8* has been associated with schizophrenia by both large-scale GWAS^47,48^ and genetic genome-wide DNA methylation analysis (brain tissues collected from 24 patients along with 24 healthy control individuals^49^). The possible involvement of this gene in the psychopathology of schizophrenia has been discussed since 2012^48^ and has been shown to involve immunologic processes, glutamatergic neurotransmission, and homeostasis of essential metals in the brain.^50,51,52,53^ In the literature,^50^ it has been hypothesized that the association between *SLC39A8* and schizophrenia may be associated with its involvement in proinflammatory immune response during brain development. Our findings highlight a negative regulation of *SLC39A8* on the nuclear factor-κ B (*NFκB*) pathway^54^ as a putative causal mechanism. The *NFκB* pathway induces the expression of proinflammatory genes (eg, cytokines),^55^ which have been associated with schizophrenic symptoms.^56^ In healthy populations, the strong association between *SLC39A8* and putamen volume may be associated with the regulatory role of *NFκB* in the growth and morphology of neurons during brain development.^57^ In patients with schizophrenia, the weakened association may be owing to dysregulation of *NFκB* in terms of gene and protein levels, and nuclear activation in brain tissues of patients.^58^ rs13107325 is a missense mutation substituting alanine (apolar) with thyronine (polar) (Ala391Thy), resulting in ZIP8-Thy391 transporting significantly less metal ion into the cell.^59^ Therefore, after the discovery of SNP rs13107325 associated with schizophrenia risk by large-scale GWAS,^47,48,50,51^ our findings indicate that molecular pathologies of schizophrenia may disrupt neuronal ion-mediated regulations in the development of putamen volume.^53^

The IMAGEN sample of 1721 homogenous 14-year-old healthy adolescents gave us an effect size (*r* = 0.21 between rs13107325 and the left putamen clusters; *r* = 0.21 between rs13107325 and the right putamen clusters) 3 times larger than that of the UKB sample of 6932 adults heterogeneously aged between 46 and 79 years (*r* = 0.06 for the left putamen clusters; *r* = 0.07 for the right putamen clusters). The genetic factors could explain up to 80% of the heritability of brain anatomy (ie, GMV), of which up to 54% could be captured by a large number of SNPs.^60^ However, percentage of variance explained by a single genetic variant was only 0.52% according to literature.^8^ In this study, the identified genetic variant explained more than 4% of the variance in the observed volumes. Such a large univariate genetic influence on the adolescent brain may be owing to less cumulative environmental impact (eg, exercises,^61^ stresses,^62^ and illnesses^63,64^) at a younger age. Perhaps the analysis of adolescents could also help explain why this novel association failed to be identified by previous large-scale meta-analyses with heterogeneous age groups.^65,66^

### Limitations

A limitation of this study is that we adopted a conservative strategy in terms of Bonferroni correction for the discovery of significant vGWAS signal. We acknowledge that this conservative procedure may give false-negative findings owing to the sample size of the discovery study. However, if we used the meta-analysis for the discovery by combining both the IMAGEN sample with the replication samples, we might have missed those associations that were significant in adolescents only. Given that the IMAGEN participants were of similar age, future imaging genetic cohorts of healthy adolescents may help us to identify more gene-brain associations with smaller effect sizes. Second, the identified brain associations of the other SNP rs7182018 were more stable across the life span, but there is no evidence to our knowledge to date that it is involved in the pathology of schizophrenia. Third, the identified gene-level eQTL result did not reach a genome-wide significance level in the UKBEC database, and rs13107325 was not associated with expression of *SLC39A8* in the GTEx (http://www.gtexportal.org). This may be partially owing to differences in the sex ratio and racial/ethnic composition between these 2 databases. Furthermore, other levels (expression of exon, junction, and transcripts) of eQTL analyses should also be conducted in the future. Animal studies to test these possible molecular mechanisms are also warranted.

## Conclusions

In summary, using an innovative method, we identified a gene that points to a potential new mechanism associated with both ion transporter and immune reaction for development of psychopathology, in particular associated with schizophrenia. Given that the major function of the *SLC39A8* gene is accessible to pharmacologic manipulation,^67,68,69^ we believe that these results are crucial for discovering novel treatment for schizophrenia.

## Notes

Supplement. eMethods 1. Genotyping and quality control in IMAGEN eMethods 2. Structural imaging and preprocessing in IMAGEN eMethods 3. Saguenay Youth Study eMethods 4. Lieber Institute for Brain Development Study eMethods 5. Three-city study eMethods 6. UK Biobank eMethods 7. Brain eQTL database eMethods 8. Voxel-wise and Genome-wide Association Study eMethods 9. Statistical meta-analyses eMethods 10. Two-sample Summary-data-based Mendelian Randomization eMethods 11. Power analysis eTable 1. Demographics of the samples eTable 2. Clusters reached a brain-wide and genome-wide significance level. eTable 3. Significant associations defined by different thresholds eTable 4. Association models with additional covariates eTable 5. Replication of associations for SNP rs7182018 eTable 6. GWAS Results in Different Sexes eTable 7. Association Results Given by Different Thresholds Using the IMAGEN Sample eTable 8. Association Results Given By Different Thresholds Using the UKB Sample eTable 9. Inputs for Meta-analysis Using the R package Metafor eTable 10. Results of Meta-analysis of the SNP Volume Associations eTable 11. A SNP to Whole-brain Replication Using the UK Biobank Sample eTable 12. Gene Expression Associations of rs7182018 eTable 13. Gene Expression Associations of rs13107325 eTable 14. LD Matrices D (R2) for Significant SNPs eFigure 1. GWAS on 4 Clusters Reached the Brain-Wide and Genome-Wide Significance Level eFigure 2. Comparison of Volumes And Histogram of t-statistics in IMAGEN Baseline Sample eFigure 3. The Association Between Putamen Volume and Schizophrenia Free of Nongenetic Confounders Given by SMR Analysis Using Each SNP as an Instrument eFigure 4. The Association Between Central Sulcus Volume and Schizophrenia Free Of Nongenetic Confounders Given by SMR Analysis Using Each SNP as an Instrument eFigure 5. Forest Plot of the Meta-analysis in the Left Central Sulcus eFigure 6. Forest Plot of the Meta-analysis in the Right Central Sulcus eFigure 7. Comparison of Volumes and Histogram of t-statistics in the SYS sample eFigure 8. Comparison of Volumes and Histogram of t-statistics in the LIBD healthy controls eFigure 9. Comparison of Volumes and Histogram of t-statistics in the LIBD patients eFigure 10. Comparison of Volumes and Histogram of t-statistics for Unaffected Siblings of Patients in the LIBD Study eFigure 11. Comparison of Volumes and Histogram of t-statistics in the UKB eFigure 12. Comparison of Volumes and Histogram of t-statistics in the 3C Sample eFigure 13. Boxplots of Gene Expression in the Frontal Cortex eFigure 14. Mappings of Putamen Clusters onto Individual Space in IMAGEN and SYS eFigure 15. Forest Plot of the Meta-analysis in the Left Putamen eFigure 16. Forest Plot of the Meta-analysis in the Right Putamen eFigure 17. GWAS Signals for GMV Using the Genome-Wide Significance Level eReferences
