# Supplementary material for: Association of a Schizophrenia-Risk Nonsynonymous Variant With Putamen Volume in Adolescents: A Voxelwise and Genome-Wide Association Study
Source: JAMA Psychiatry. 2019 Jan 16;76(4):435–45. doi: 10.1001/jamapsychiatry.2018.4126 (PMC6450291; doi:10.1001/jamapsychiatry.2018.4126)
Supplement: Supplement. — eMethods 1. Genotyping and quality control in IMAGEN eMethods 2. Structural imaging and preprocessing in IMAGEN eMethods 3. Saguenay Youth Study eMethods 4. Lieber Institute for Brain Development Study eMethods 5. Three-city study eMethods 6. UK Biobank eMethods 7. Brain eQTL database eMethods 8. Voxel-wise and Genome-wide Association Study eMethods 9. Statistical meta-analyses eMethods 10. Two-sample Summary-data-based Mendelian Randomization eMethods 11. Power analysis eTable 1. Demographics of the samples eTable 2. Clusters reached a brain-wide and genome-wide significance level. eTable 3. Significant associations defined by different thresholds eTable 4. Association models with additional covariates eTable 5. Replication of associations for SNP rs7182018 eTable 6. GWAS Results in Different Sexes eTable 7. Association Results Given by Different Thresholds Using the IMAGEN Sample eTable 8. Association Results Given By Different Thresholds Using the UKB Sample eTable 9. Inputs for Meta-analysis Using the R package Metafor eTable 10. Results of Meta-analysis of the SNP Volume Associations eTable 11. A SNP to Whole-brain Replication Using the UK Biobank Sample eTable 12. Gene Expression Associations of rs7182018 eTable 13. Gene Expression Associations of rs13107325 eTable 14. LD Matrices D (R2) for Significant SNPs eFigure 1. GWAS on 4 Clusters Reached the Brain-Wide and Genome-Wide Significance Level eFigure 2. Comparison of Volumes And Histogram of t-statistics in IMAGEN Baseline Sample eFigure 3. The Association Between Putamen Volume and Schizophrenia Free of Nongenetic Confounders Given by SMR Analysis Using Each SNP as an Instrument eFigure 4. The Association Between Central Sulcus Volume and Schizophrenia Free Of Nongenetic Confounders Given by SMR Analysis Using Each SNP as an Instrument eFigure 5. Forest Plot of the Meta-analysis in the Left Central Sulcus eFigure 6. Forest Plot of the Meta-analysis in the Right Central Sulcus eFigure 7. Comparison of Volume [file jamapsychiatry-76-435-s001.pdf]

# Supplementary Online Content

Luo Q, Chen Q, Wang W, et al; the IMAGEN Consortium. Association of a schizophrenia-risk nonsynonymous variant with putamen volume: a voxelwise and genome-wide association study. *JAMA Psychiatry.* Published online January 16, 2018. doi:10.1001/jamapsychiatry.2018.4126

**eMethods 1.** Genotyping and quality control in IMAGEN
**eMethods 2.** Structural imaging and preprocessing in IMAGEN
**eMethods 3.** Saguenay Youth Study (SYS)
**eMethods 4.** Lieber Institute for Brain Development (LIBD) Study
**eMethods 5.** Three-city (3C) study
**eMethods 6.** UK Biobank
**eMethods 7.** Brain eQTL database
**eMethods 8.** Voxel-wise and Genome-wide Association Study (vGWAS)
**eMethods 9.** Statistical meta-analyses
**eMethods 10.** Two-sample Summary-data-based Mendelian Randomization
**eMethods 11.** Power analysis
**eTable 1.** Demographics of the samples
**eTable 2.** Clusters reached a brain-wide and genome-wide significance level.
**eTable 3.** Significant associations defined by different thresholds
**eTable 4.** Association models with additional covariates
**eTable 5.** Replication of associations for SNP rs7182018
**eTable 6.** GWAS Results in Different Sexes
**eTable 7.** Association Results Given by Different Thresholds Using the IMAGEN Sample
**eTable 8.** Association Results Given By Different Thresholds Using the UKB Sample
**eTable 9.** Inputs for Meta-analysis Using the R package Metafor
**eTable 10.** Results of Meta-analysis of the SNP Volume Associations
**eTable 11.** A SNP to Whole-brain Replication Using the UK Biobank Sample
**eTable 12.** Gene Expression Associations of rs7182018
**eTable 13.** Gene Expression Associations of rs13107325.
**eTable 14.** LD Matrices D' (R2) for Significant SNP's
**eFigure 1.** GWAS on 4 Clusters Reached the Brain-Wide and Genome-Wide Significance Level
**eFigure 2.** Comparison of Volumes And Histogram of t-statistics in IMAGEN Baseline Sample
**eFigure 3.** The Association Between Putamen Volume and Schizophrenia Free of Nongenetic Confounders Given by SMR Analysis Using Each SNP as an Instrument
**eFigure 4.** The Association Between Central Sulcus Volume and Schizophrenia Free Of Nongenetic Confounders Given by SMR Analysis Using Each SNP as an Instrument
**eFigure 5.** Forest Plot of the Meta-analysis in the Left Central Sulcus
**eFigure 6.** Forest Plot of the Meta-analysis in the Right Central Sulcus
**eFigure 7.** Comparison of Volumes and Histogram of t-statistics in the SYS sample
**eFigure 8.** Comparison of Volumes and Histogram of t-statistics in the LIBD healthy controls
**eFigure 9.** Comparison of Volumes and Histogram of t-statistics in the LIBD patients
**eFigure 10.** Comparison of Volumes and Histogram of t-statistics for Unaffected Siblings of Patients in the LIBD Study
**eFigure 11.** Comparison of Volumes and Histogram of t-statistics in the UKB
**eFigure 12.** Comparison of Volumes and Histogram of t-statistics in the 3C Sample
**eFigure 13.** Boxplots of Gene Expression in the Frontal Cortex

© 2019 Luo Q et al. *JAMA Psychiatry.*

**eFigure 14.** Mappings of Putamen Clusters onto Individual Space in IMAGEN and SYS
**eFigure 15.** Forest Plot of the Meta-analysis in the Left Putamen
**eFigure 16.** Forest Plot of the Meta-analysis in the Right Putamen
**eFigure 17.** GWAS Signals for GMV Using the Genome-Wide Significance Level (P < 5e-8)
**eReferences**

This supplementary material has been provided by the authors to give readers additional information about their work.

© 2019 Luo Q et al. *JAMA Psychiatry.*

**eMethod 1. Genotyping and quality control in IMAGEN**

DNA purification and genotyping were performed by the Centre National de Génotypage in Paris. DNA was purified from whole-blood samples (~10ml) preserved in BD V acutainer EDTA tubes (Becton, Dickinson and Company, Oxford, UK) using the Gentra Puregene Blood Kit (Qiagen, Manchester, UK) according to the manufacturer's instructions. A total of 705 and 1,382 individuals were genotyped with the Illumina (Little Chesterford, UK) Human610-Quad Beadchip (582,982 SNPs) and Illumina Human660-Quad Beadchip (557,124 SNPs), respectively. Both of them were based on the same Illumina HumanHap550 Genotyping BeadChip with varied additional probes, and therefore they have most of their SNPs (551,141 SNPs) in common, with the same rsID and location based on assembly hg18. Before merging, strand flipping was conducted to make sure that the same strand was used to recode the same genotypes from both platforms. Genotypes from both platforms were then combined through software PLINK where platform-specific SNPs were removed. The following procedures of quality control were conducted. Single-nucleotide polymorphisms (SNPs) with call rates <95%, minor allele frequency <5%, deviation from the Hardy–Weinberg equilibrium ($P \leq 1 \times 10^{-3}$) and nonautosomal SNPs were excluded from the analyses. Individuals with excessive missing genotypes (failure rate >5%) were also excluded. Population homogeneity was examined with the Structure software with a cut-off of 0.95 using HapMap populations as reference groups. Individuals with divergent ancestry (from Utah residents with ancestry from northern and western Europe) were excluded. Identity-by-state clustering and the classic multidimentional scaling with visual inspection on the first two dimensions were used to estimate cryptic relatedness for each pair of individuals using the PLINK software and closely related individuals (n=2) were eliminated from the subsequent analysis. We applied principal component analysis to remove remaining outliers, defined as individuals located at more than four s.d. of the mean principal component analysis scores on one of the first 20 dimensions[1]. After the quality control measures, we obtained a total of 466, 114 SNPs in 1834 individuals.

All adult participants provided written informed consent after information on the research procedures by each cohort study. For adolescent participants, all participants' parents provided written informed consent after information on the research procedures and adolescents provided their assent after written information.

© 2019 Luo Q et al. *JAMA Psychiatry.*

**eMethod 2. Structural imaging and preprocessing in IMAGEN**

Structural MRI was performed on 3 Tesla scanners from three manufacturers (Siemens: 5 sites; Philips: 2 sites; and General Electric: 2 sites). High-resolution anatomical MRIs (T1-weighted) were obtained using a three-dimensional magnetization prepared gradient-echo sequence based on the ADNI protocol (http://adni.loni.ucla.edu/research/protocols /mri-protocols/; modified for the IMAGEN study to give a $1.1\times1.1\times1.1mm^3$ voxel size; repetition time = 2300ms, echo time = 2.8ms, flip angle = 8°; 240×256×160 matrix.).

All data were preprocessed in SPM8 (http://www.fil.ion.ucl.ac.uk/spm/) using the VBM8 toolbox with default settings, including the usage of high-dimensional spatial normalization with an already integrated Dartel template in MNI space. All images were subjected to nonlinear modulations and corrected for each individual head size. Images were then smoothed with an 8 mm full-width at half-maximum Guassian kernel with the resulting voxel size $1.5mm^3$. Two covariates were calculated for grey-matter volume, the estimated total intracranial volume (eTIV) and the whole-brain volume (eWBV). The eTIV was estimated by the summation of the grey matter, white matter, and CSF volumes in native space. The eWBV was the portion of the raw grey and white matter volume in the eTIV. The automated anatomical labeling (AAL) atlas was employed to exclude the voxels outside the grey matter.

© 2019 Luo Q et al. *JAMA Psychiatry.*

**eMethod 3. Saguenay Youth Study (SYS)**

The SYS sample was recruited from a population with the known genetic-founder effect living in the Saguenay–Lac-Saint-Jean (SLSJ) region of Quebec, Canada. All participants are White Caucasians of French descent [2] (http://www.saguenayyouth-study.org).

**Genetic data** All adolescents were genotyped with the Illumina Human610-Quad BeadChip (610K SNPs) or Illumina HumanOmniExpress BeadChip (700k SNPs). Imputations were used to combine the two platforms. We employed an imputation protocol developed by the ENIGMA Working Group, and imputed genotypes using a reference file created by the ENIGMA2 Genetics Support Team. This reference file is based on the 1kG Project (phase 1, release v3; ~41M SNPs)[3] and includes only ~13M SNPs that are polymorphic in Caucasians and have been observed more than once in European samples. Haplotype phasing was performed with SHAPEIT[4] using an overlapping subset of 313,653 post-quality-control SNPs that were present on both genotyping platforms and the above reference panel. Imputation was conducted on the phased data with IMPUTE2[5]. Markers with low imputation quality (information score <0.5) or low minor allele frequency (<0.01) were removed. Both SNPs rs13107325 and rs7182018 were genotyped.

**Structural MRI Data** Three dimensional structural magnetic resonance (MR) images of the brain were acquired on a Phillips 1.0-T superconducting magnet using three dimensional (3D) radio frequency (RF)-spoiled gradient-echo scan with 140–160 slices, an isotropic resolution of 1 mm, a repetition time (TR) of 25 ms, an echo time (TE) of 5ms and a flip angle of 30°.

**Image Analysis** First, the native T1-weighted images were transformed non-linearly into standardized stereotaxic space, using the average brain computed from our population (SYS333) as the registration target; the SYS333 was aligned with the MNI-305 template, which was in turn aligned with the co-planar stereotaxic atlas. Local differences between the subject's brain and the template were captured by the deformation field estimated through the non-linear registration. The next step involved the segmentation of brain tissue into GM, WM and cerebrospinal fluid (CSF) maps; this was done using contrast from the T1-weighted acquisitions. This step produced three sets of binary 3-D images of WM, GM and CSF. The template brain also contained information concerning anatomical boundaries, which were "projected" onto each subject's brain in native space using the corresponding deformation field, which was then fine-tuned by combining it with the tissue classification map. The combination of the atlas-based lobar boundaries with the tissue maps provided automatic estimates of lobar volumes of GM. The binary tissue-classified GM brain maps, transformed to the SYS333 space using the non-linear registration, were then blurred using a Gaussian smoothing kernel of 10 mm full-width at half-maximum in order to create 3-D density maps of GM for voxel-wise analyses. The mask of those four clusters defined in the IMAGEN sample was mapped from the standard space to the native space through a non-linear registration. Finally, a quality check was performed to identify any potential problems with either the registration or classification steps. This quality check consisted of identifying clear failures of non-linear registration and/or tissue classification by inspecting the original (raw) MR images together with the registered (with SYS333 template) and classified images in all subjects.

**Association Study** After quality control for both genetic data and image data, we had 971 adolescents in the SYS sample. The association of the SNP and the grey-matter volumes were analyzed, and Sex, Age, Age×Age, eTIV, and handedness were used as covariates. The 95% confidence interval of the statistics were given by 3000 bootstraps, and the boxplots for comparison of brain volumes between genotypes were presented in eFigure 7.

© 2019 Luo Q et al. *JAMA Psychiatry.*

**eMethod 4. Lieber Institute for Brain Development (LIBD) Study**

This study was conducted by the Lieber Institute for Brain Development (LIBD), US (http://www.libd.org/). All subjects are Caucasian. After quality control and preprocessing, the LIBD sample includes 272 healthy participants (mean age 32 years), 157 chronic treated patients with schizophrenia (mean age 35 years) and 149 unaffected siblings (mean age 37 years), who were recruited as part of the Clinical Brain Disorders Branch Sibling Study (National Institutes of Health Study ID NCT00001486). All participants were right handed. For detailed demographic information and inclusion/exclusion criterion of this clinical study we refer to our previous publication [6].

**Genetic Data** For LIBD samples, genotyping was done using Illumina BeadChips (510/610/660/2.5). Quality control was performed using PLINK (version 1.07; http://pngu.mgh.harvard.edu/purcell/plink/). Participants with missing rate higher than 2% and extreme heterozygosity values (±3 SD) were removed. SNPs with missing rate higher than 2% and difference in missingness between cases and controls > 0.02 were removed. SNPs were also excluded if they failed Hardy-Weinberg equilibrium test ($P < 10^{-6}$ in controls or $P < 10^{-10}$ in cases) and if they have minor allele frequency less than 1%. Pre-phasing was done before imputation using SHAPEIT, and imputation was done using IMPUTE2 with 1000 genome as reference panel.

**Structural MRI Data** Three-dimensional structural MRI scans were acquired on a 1.5-T GE scanner (GE Medical Systems, Milwaukee, Wisconsin) using a T1-weighted spoiled gradient recalled sequence (repetition time, 24 milliseconds; echo time, 5 milliseconds; number of excitations, 1; flip angle, 45°; matrix size, 256x256; field of view, 24x24 cm), with 124 sagittal slices (0.94x0.94x1.5mm resolution). Images were processed by VBM8 in SPM8 following the same procedure as described above for the IMAGEN sample. The mask defined by the association result in the IMAGEN sample of those four significant clusters in the standard MNI space was then applied to the normalized brain images to extract the corresponding grey-matter volumes.

**Association analysis** The association of the SNP and the grey-matter volumes were analyzed in R, and Sex, Age, Age×Age, IQ, eTIV and eWBV were used as covariates. The 95% confidence interval of the statistics were given by 3000 bootstraps, and the boxplots for comparison of brain volumes between genotypes were presented in eFigures 8-10.

**eMethod 5. Three-city (3C) study**

This study aims to evaluate the risk of dementia and cognitive impairment attributable to vascular factors. It is conducted in three cities of France: Bordeaux, Dijon and Montpellier (http://www.three-city-study.com/). In this analysis, we used the healthy control individuals in Bordeaux, France as both genetic data and neuroimaging data were available at this site.

**Genetic data** Successful genome-wide genotyping was performed on the Illumina Human610-Quad Beadchip. The quality control was performed by the Pharnext, France. After a data set pruning, the filtering of samples and exclusion of subjects was based on the following criteria: individual call rate> 5%, inbreeding coefficient >0.2, sex mismatch, identical by descent >0.1875 and detection of outliers using principal component analysis (PCA) by EIGENSOFT. There were 6467 subjects remained. Then, the filtering of SNPs was performed based on: SNP call rate>5 %, heterozygous number <40, minor allele frequency <0.0017 and Hardy-Weinberg disequilibrium $p<8.6^{-08}$ in controls. After filtering, 542 058 SNPs (93%) were left for association analyses.

**Structural MRI Data** Structural MRI was performed on 1.5-Tesla scanner with fast 3-dimensioal spoiled gradient-echo T1-weighted axial acquisitions with 2 excitations. Data preprocessing was performed by Laboratoire de recherche en neuroimagerie, CHUV Lausanne. All data used here have gone through visual quality check. The grey matter was subsequently normalized to MNI space using the Dartel method. The smoothing kernel has FWHM 8mm. The resulting voxel size is $1.5mm^3$. Images were processed by VBM8 in SPM8 following the same procedure as described above for the IMAGEN sample. The mask defined by the association result in the IMAGEN sample of those four significant clusters in the standard MNI space was then applied to the normalized brain images to extract the corresponding grey-matter volumes.

**Association analysis** Among all 634 subjects after the quality control, 515 subjects had genetic data available. Therefore, 515 subjects were used for the replication of the association between SNP and GMV. The association of the SNP and the grey-matter volumes were analyzed by Plink2 using linear regression. Sex, age, age×age, education, eTIV and eWBV were used as covariates in association analysis. The 95% confidence interval of the statistics were given by 3000 bootstraps, and the boxplots for comparison of brain volumes between genotypes were presented in eFigure 12.

© 2019 Luo Q et al. *JAMA Psychiatry.*

**eMethod 6. UK Biobank**

UK Biobank is a prospective epidemiological study with multimodal population brain imaging and genetic information (http://www.ukbiobank.ac.uk/) [7].

**Structural MRI Data** In the Februray 2017 release, a single scanner dedicated to UKB imaging in Cheadle Manchester. The scanner is a standard Siemens Skyra 3T running VD13A SP4 (as of October 2015), with a standard Siemens 32-channel RF receive head coil. The imaging protocol is as follows: Resolution: 1×1×1 mm Field-of-view: 208×256×256 matrix Duration: 5 minutes 3D MPRAGE, sagittal, in-plane acceleration iPAT=2, prescan-normalise For more details we refer to the official document for neuroimaging of the UKB (http://biobank.ctsu.ox.ac.uk /crystal/docs/brain_mri.pdf). To be consistent, we followed exactly the same pre-processing procedure as the IMAGEN study using the VBM8. In total, we were able to successfully process T1 images of 9 880 subjects, and extracted the grey matter volumes of the predefined clusters by the discovery sample.

**Genetic data** Genotype data are available for all 500,000 participants in the UKB cohort. Genotyping was performed using the Affymetrix UK BiLEVE Axiom array on an initial 50,000 participants; the remaining 450,000 participants were genotyped using the Affymetrix UK Biobank Axiom® array. The two arrays are extremely similar (with over 95% common content). Among those participants with neuroimaging data been successfully pre-processed using the VBM8, 6 932 subjects had also the genotype information at both SNPs [rs13107325 (genotyped, MAF = 0.07) and rs7182018 (imputed by IMPUTE2, MAF = 0.09, INFO score = 0.9961)] after imputation from the March 2018 release. All subjects in this subset were estimated to have recent British ancestry and have no more than 10 putative third-degree relatives in the kinship table using the sample quality control information provided centrally by UKB (using the variables of *white.British.ancestry.subset* and *excess.relatives* in the file *ukb_sqc_v2.txt*). For more details we refer to the official document for genetic data of the UKBk (http://www.ukbiobank.ac.uk/scientists-3/genetic-data/).

**Association analysis** In total, 6932 subjects were used for the replication of the association between SNP and GMV. The association of the SNP and the grey-matter volumes were analyzed by linear regression model. Sex, age (we used the age when the participants were actually scanned, which was the third instance of the date when the participants attended assessment centre recorded in Data-Field 21003 minus the date of birth recorded in Data-Field 33), age×age, eTIV were used as covariates in association analysis. We also replicated the findings by including either BMI ($t_{6870}$ = 14.87/16.42, p = $1.59\times10^{-49}$/$8.61\times10^{-60}$ for the clusters in the left/right central sulcus; $t_{6870}$ = 4.85/6.49, p = $6.44\times10^{-7}$/$4.69\times10^{-11}$ for the clusters in the left/right putamen) or eWBV ($t_{6885}$ = 15.05/16.52, p = $1.03\times10^{-50}$/$6.89\times10^{-51}$ for the clusters in the left/right central sulcus; $t_{6885}$ = 3.74/5.58, p = $9.34\times10^{-5}$/$1.24\times10^{-8}$ for the clusters in the left/right putamen) as additional covariates. The 95% confidence intervals of the statistics were given by 3000 bootstraps, and the boxplots for comparison of brain volumes between genotypes were presented in eFigure 11.

© 2019 Luo Q et al. *JAMA Psychiatry.*

**eMethod 7. Brain eQTL database**

A database for eQTL (expression Quantitative Trait Loci) in the brain was made publicly available by the UK Brain Expression Consortium (UKBEC[8]). The central nervous system tissues originating from 134 neuropathologically normal individuals was collected by the Medical Research Council (MRC) Sudden Death Brain and Tissue Bank, Edinburgh, UK, and the Sun Health Research Institute (SHRI) an affiliate of Sun Health Corporation, USA. From each individual, up to 10 brain regions were analyzed, including cerebellar cortex (CRBL), frontal cortex (FCTX), hippocampus (HIPP), medulla (specifically inferior olivary nucleus, MEDU), occipital cortex (specifically primary visual cortex, OCTX), putamen (PUTM), substantia nigra (SNIG), thalamus (THAL), temporal cortex (TCTX) and intralobular white matter (WHMT). The Affymetrix Exon 1.0ST Arrays were used to measure the RNA transcripts, and all arrays were pre-processed and log2 transformed following the standard protocol. Genomic DNA was extracted from sub-dissected samples of human post-mortem brain tissue, and all samples were genotyped on the Illumina Infinium Omni1-Quad BeadChip with a custom genotyping array. The expression QTL for each expression profile against every genetic marker in MatrixEQTL, and subsequent analyses were conducted in R. All gene expression, genotype, and eQTL results were free to access through a website http://www.braineac.org/.

First, as the SNP rs13107325 is a missense variant change Als391 to Thr391 in the ZIP8 protein, and this substitution is likely to cause structural change in a α-helical transmembrane domain of the ZIP8 protein. Therefore, we first tested whether this SNP was associated with expression of the gene SLC39A8 in the putamen. We identified a significant association between the SNP rs13107325 and gene expression of SLC39A8, particularly measured by the exon-specific probe Affymetrix ID 2779840, which was a probe of a transcript cluster (ID 2779823) of SLC39A8.

Second, to test whether such association was tissue specific to putamen among other brain regions and whether this SNP also has the cis effects on nearby genes, we second tested such association against 10 brain tissues available in the UKBEC database and 6 genes within ±1Mb of the location of rs13107325. For this extended search, we adopted a threshold of 0.0008 (= 0.05/10/6, corrected for 10 types of brain tissues and 6 genes).

Rs7182018 is located on gene RP11-624L4.1 (GENCODE V28), which has been suggested to be a lincRNA but its function is yet to be characterized. Using the UKBEC database (GG=4, GA=18, AA=112, MAF = 9.7%), we had queried the eQTL for two genes THBS1 and C15orf52 next to the gene RP11-624L4.1, and found evidences suggesting cis-eQTL effects in tissues from the frontal cortex ($t_{125} = 3.9361$, $p = 0.00014$ for THBS1, and $t_{125} = 3.7989$, $p = 0.00023$ for C15orf52; eTable 12 for queried p-value and eFigure 13 for boxplots comparing gene expressions among genetic groups).

© 2019 Luo Q et al. *JAMA Psychiatry.*

**eMethod 8. Voxel-wise and Genome-wide Association Study (vGWAS)**

**Association analysis** Firstly, we did the genome-wide association study for the whole brain by using the grey-matter volumes of each voxel as a continuous trait. The AAL (automatically anatomic labeling[9]) template was used to restrict our analysis in the grey matter of the brain. Therefore, the whole brain was divided into 438 145 voxels. A significant association was identified if a cluster with more than 217 ($\approx 4/3 \times \pi \times (3.3970 \times 1.645)^3 / 1.5^3$ falling into the 90% confidence interval of the smoothing kernel) voxels (~0.7ml) had the p values of the associations all survived a Bonferroni correction at the brain-wide and genome-wide significant level ($p < 2.4483\times10^{-13} = 0.05$ / 438 145 [n of voxels] / 466 114 [n of SNPs]). As the statistical threshold of significance can be given by various criterions, we also tried one more stringent threshold ($p < 1.1412\times10^{-13} = 5\times10^{-8}$ [genome-wide significance level ] / / 438 145 [n of voxels] ) and one less stringent threshold ( $\alpha_p = 1-(1-5\times10^{-8})^{1/M_{eff}} = 1.9417\times10^{-11}$), where $M_{eff} = 2575$ was the effective number of independent tests (https://neurogenetics.qimrberghofer.edu.au /SNPSpDlite/[10]). The results were listed in eTable 3. As expected, the higher threshold gave smaller clusters while the lower threshold gave larger clusters. Both of them were at the same locations as the clusters defined by the Bonferroni threshold. The GWAS results at the cluster level were listed in eTable 7 for IMAGEN baseline sample and eTable 8 for UK Biobank sample.

Secondly, the clusters identified by the brain-wide and genome-wide association analysis defined regions of interest. To control the possible confounding factors, we used 14 covariates during the association study, including sex, handedness, site (7 dummy variables for 8 sites), eTIV, and first four principal components from multidimensional scaling analysis[11]. Excluding the subjects with any missing values in the genetic data, volume, or covariates, we had 1 721 subjects left for the GWAS. Since rs13107325 had been associated with BMI[12], we considered BMI as an additional covariate to see if the observed association remains. To further conditioning out the potential confounding effects, we also tried to include the age, perceptual reasoning IQ, verbal comprehension IQ, and puberty as further covariates to see if the identified association holds (eTable 4). A mask of these significant clusters were generated, and applied to other independent samples to verify our findings. Followed the literature[13], the percentage variance explained by each genome-wide significant SNP was estimated after accounting for covariate effects.

Two clusters in bilateral putamen were reported in the main text, and the other two clusters in the central sulcus were as follows: the MNI coordinate of the peak voxel at the left was (-49.5, -7.5, 30) and the association was estimated as $b = 1.78\times10^{-9}$ and $p=2.75\times10^{-22}$; at the right, the association of the peak voxel (54, -4.5, 19.5) was $b = 1.50\times10^{-9}$ and $p=6.27\times10^{-26}$.

To facilitate the replications and the behavioural analysis, we used the volumes of the identified clusters instead of the volume of each voxel in the following analyses. The grey matter volume of a cluster was calculated by summing over all voxels within that cluster[14]. As expected, the association between the SNPs and the volumes of these identified clusters were still significant ($b>0$, $p<10^{-17}$, eFigure 1 and Table 1; eTable 5). We first made a mask for those four significant clusters identified using the IMAGEN sample (*i.e.*, two clusters in the bilateral putamen and two clusters in the bilateral central sulcus). Second, by applying this mask labelling 4 regions-of-interest (ROI) to structural MRI data from each replicating cohort, we estimated the grey matter volume (GMV) for each ROI. Third, we calculated the associations between SNP rs13107325 and GMV of each of these two putamen ROI's, and between SNP rs7182018 and GMV of each of these two central sulcus ROI's.

As IMAGEN is a longitudinal cohort, we also tested whether our findings in the IMAGEN sample at age 14 years remained significant 5 years later when these participants turned to age 19 years. Among 869 19-year-old subjects in the follow-up sample of the IMAGEN study, we confirmed that SNP rs13107325 remained significantly associated with both the clusters ($t_{854} = 5.86/6.26$, 95% CI = 3.89—7.74 / 4.33—8.57, $p = 3.33\times10^{-9}/3.08\times10^{-10}$, variance explained = 3.86%/4.38% in the left [1.78±0.30ml] /right [0.69±0.09ml] putamen; n = 869).

**eMethod 9. Statistical meta-analyses**

Putting together all 5 samples, including the IMAGEN sample at the baseline, the SYS sample, the LIBD healthy control sample, the UKB sample, and 3C sample, we conducted a meta-analysis of the identified associations with a fixed effect model. The analyses were implemented by the R package *metafor* http://www.metafor-project.org/ and the forest plots were also generated by this package.

Meta-analysis of all samples confirmed the identified associations across the life span (n =10411; Z = 10.12/10.79, p = $4.57\times10^{-24}$/$3.74\times10^{-27}$ in the left/right putamen; Z = 18.19/10.10, p = $5.95\times10^{-74}$/$2.32\times10^{-81}$ in the left/right central sulcus; eTables 9-10 for more statistics and eFigures 5-6 and 15-16 for the forest plots of these meta-analyses).

© 2019 Luo Q et al. *JAMA Psychiatry.*

**eMethod 10. Two-sample Summary-data-based Mendelian Randomization**

To test whether the pleiotropic associations of SNP rs13107325 with both grey matter volume of the putamen cluster and the risk for schizophrenia, we conducted a 2-sample summary-data-based Mendelian randomization (SMA) analysis as proposed in the literature[15]. In this analysis, we considered SNP rs13107325 as the instrument variable, GMV of the right putamen volume as the exposure variable (the current vGWAS in IMAGEN), and schizophrenia as the outcome variable (GWAS by PGC2[16]). This analysis was conducted by a web-based version of the MR-Base (http://www.mrbase.org), which is a platform integrating a curated database of complete GWAS results[17]. A significant result given by the SMR suggests a significant pleiotropic associations of both exposure variable and outcome variable with the instrument variable (SNP), and furthermore there might also be a causal effect of the exposure on the outcome, i.e., a vertical pleiotropic association, but inferring causality using observational data must be interpreted with great caution[15].

As vGWAS signal might indicate a real effect from the genetic variants in a LD block of the indicator SNP, we submitted the SMR results of all SNPs within ±1M bp of the indicating SNP to Locus Zoom (http://locuszoom.org/) to plot the MR results together with recombination rate. With these figures, we could check whether there was any significant effect of the nearby SNPs that are less likely to be separated from the indicating SNP due to recombination. It is also recommended that using multiple independent instrumental variables may provide further support on the causal relationship between exposure and outcome[17]. Using the standard genome-wide significance level ($p<5e-8$), we identified more SNPs associated with either putamen clusters or central sulcus clusters that could be used as instrumental variables (eFigure 17). However, we found that these SNPs were in the same LD block with either SNPs rs13107325 or rs7182018 (eTable 14). Therefore, we reported the SMR results for the SNPs with the strongest association only.

© 2019 Luo Q et al. *JAMA Psychiatry.*

**eMethod 11. Power analysis**

In the LIBD study, we had 272 healthy controls. We estimated the partial correlation between genotype of rs13107325 and grey matter volume of the right putamen cluster was 0.3117 using age, age^2, IQ, sex, TIV and WBV as covariates. Setting Test family as t tests, Statistical test as Linear multiple regression: Fixed model, single regression coefficient, Type of power analysis: A priori: Compute required sample size—given α, power, and effect size in G*Power (http://www.gpower.hhu.de/en.html), we estimated the required sample size as 102 for 95% power assuming a 5% significance level and a one-side test. Therefore, the LIBD study provided us adequate sample sizes (157 patients and 149 unaffected siblings) for the current analysis.

© 2019 Luo Q et al. *JAMA Psychiatry.*

**eTable 1. Demographics of the samples.**

| Role | Study | Age(y) | Subjects (Female) | MAF | | PANSS score | | |
|---|---|---|---|---|---|---|---|---|
| | | | | rs13107325 | rs7182018 | Positive | Negative | General |
| Discovery | IMAGEN Baseline | 14.44±0.41 | 1 721 (873) | 0.07 | 0.09 | / | / | / |
| Longitudinal | IMAGEN Follow-up | 19.00±0.73 | 869 (445) | 0.06 | 0.08 | / | / | / |
| Replication | SYS | 15.03±1.84 | 971 (504) | 0.06 | 0.1 | / | / | / |
| | 3C | 77.48±5.12 | 515 (293) | 0.06 | 0.09 | / | / | / |
| | UKB | 62.64±7.41 | 6 932 (3584) | 0.07 | 0.09 | / | / | / |
| | LIBD HC | 31.92±9.50 | 272 (116) | 0.09 | 0.07 | / | / | / |
| Clinical | LIBD SZ | 34.82±9.91 | 157 (35) | 0.08 | 0.08 | 12.5±5.48 | 17.97±9.73 | 26.51±9.9 |
| | LIBD SB | 36.60±9.44 | 149 (85) | 0.10 | 0.07 | / | / | / |

**eTable 2. Clusters reached a brain-wide and genome-wide significance level.**

Significant clusters defined by the association study after Bonferroni correction ($p<2.45\times10^{-13}$) and cluster size correction (>217 voxels falling into the 90% confidence interval of the smoothing kernel, ~0.7ml). The betas and p-values of these associations were listed for the peak voxels. MAF is the frequency rate of the minor allele A2. 'A1 A1' coded as 0, 'A1 A2' coded as 1, and 'A2 A2' coded as 2. The 'L.PUT' is short for the left putamen, 'R.PUT' for the right putamen, 'L.CEN' for the left central sulcus, and the 'R.CEN' for the right central sulcus.

| **Marker** | **A1** | **A2** | **MAF** | **Functional Consequence** | **Gene** | **Chr** | **Cluster** | | | | | $t_{1705}$ | **p-value** |
|---|---|---|---|---|---|---|---|---|---|---|---|---|---|
| | | | | | | | Name | Peak (MNI) | | | Size (voxels) | | |
| | | | | | | | | X | Y | Z | | | |
| rs13107325 | C | T | 0.07 | missense | SLC39A8 | 4 | L.PUT | -25.5 | 9 | 0 | 639 | 9.28 | $5.11\times10^{-20}$ |
| | | | | | | | R.PUT | 33 | -7.5 | 0 | 263 | 9.01 | $5.38\times10^{-19}$ |
| rs7182018 | A | G | 0.09 | \ | \ | 15 | L.CEN | -49.5 | -7.5 | 30 | 710 | 9.85 | $2.75\times10^{-22}$ |
| | | | | | | | R.CEN | 54 | -4.5 | 19.5 | 452 | 10.71 | $6.27\times10^{-26}$ |

**eTable 3. Significant associations defined by different thresholds.**

Significant clusters defined by the association study according to two alternative criterions for multiple comparisons and cluster size correction (>217 voxels falling into the 90% confidence interval of the smoothing kernel, ~0.7ml). The betas and p-values of these associations were listed for the peak voxels. MAF is the frequency rate of the minor allele A2. 'A1 A1' coded as 0, 'A1 A2' coded as 1, and 'A2 A2' coded as 2. The 'L.PUT' is short for the left putamen, 'R.PUT' for the right putamen, 'L.CEN' for the left central sulcus, and the 'R.CEN' for the right central sulcus.

| Threshold | Marker | A1 | A2 | MAF | Functional Consequence | Gene | Chr | Cluster | | | | | T | p-value |
|---|---|---|---|---|---|---|---|---|---|---|---|---|---|---|
| | | | | | | | | Name | Peak (MNI) | | | Size | | |
| | | | | | | | | | X | Y | Z | (voxels) | | |
| $1.1412\times10^{-13}$ | rs13107325 | C | T | 0.07 | missense | SLC39A8 | 4 | L.PUT | -25.5 | 9 | 0 | 600 | 9.28 | $5.11\times10^{-20}$ |
| | | | | | | | | R.PUT | 33 | -7.5 | 0 | 227 | 9.01 | $5.38\times10^{-19}$ |
| | rs7182018 | A | G | 0.09 | \ | \ | 15 | L.CEN | -49.5 | -7.5 | 30 | 672 | 9.85 | $2.75\times10^{-22}$ |
| | | | | | | | | R.CEN | 54 | -4.5 | 19.5 | 425 | 10.71 | $6.27\times10^{-26}$ |
| $1.9417\times10^{-11}$ | rs13107325 | C | T | 0.07 | missense | SLC39A8 | 4 | L.PUT | -25.5 | 9 | 0 | 924 | 9.28 | $5.11\times10^{-20}$ |
| | | | | | | | | R.PUT | 33 | -7.5 | 0 | 501 | 9.01 | $5.38\times10^{-19}$ |
| | rs7182018 | A | G | 0.09 | \ | \ | 15 | L.CEN | -49.5 | -7.5 | 30 | 1012 | 9.85 | $2.75\times10^{-22}$ |
| | | | | | | | | R.CEN1 | 54 | -4.5 | 19.5 | 648 | 10.71 | $6.27\times10^{-26}$ |

**eTable 4. Association models with additional covariates.**
P values were given by one-tailed test. The associations were estimated for the volumes of the significant clusters identified by our vGWAS. The volume of a cluster was calculated by adding up the volume of each voxel within that cluster. Additional covariates were added into the model which had already included 14 covariates as specified in the eMethod for vGWAS. This analysis was conducted using the discovery sample, i.e., IMAGEN baseline.

| | | **Association** | | | |
|---|---|---|---|---|---|
| **Additional covariates (n)** | statistics | rs13107325-left PUT | rs13107325-right PUT | rs7182018-left CEN | rs7182018-right CEN |
| eWBV (1721) | p | 2.02E-19 | 6.80E-19 | 7.19E-24 | 3.40E-26 |
| | t | 9.0428 | 8.9029 | 10.1561 | 10.6982 |
| vIQ and pIQ (1527) | p | 1.53E-16 | 3.06E-17 | 2.38E-18 | 3.00E-19 |
| | t | 8.264 | 8.462 | 8.7693 | 9.0113 |
| BMI (1612) | p | 6.36E-18 | 6.09E-18 | 2.86E-20 | 6.13E-21 |
| | t | 8.6465 | 8.6516 | 9.2723 | 9.4441 |
| age (1707) | p | 1.70E-18 | 1.66E-19 | 5.63E-22 | 1.66E-22 |
| | t | 8.7966 | 9.0662 | 9.6969 | 9.828 |
| puberty (1700) | p | 6.40E-19 | 4.82E-20 | 1.31E-21 | 1.46E-21 |
| | t | 8.9113 | 9.2071 | 9.6061 | 9.5947 |

**eTable 5. Replication of associations for SNP rs7182018.**

P values were given by one-tailed test. The associations were estimated for the volumes of the significant clusters identified by our vGWAS. The volume of a cluster was calculated by adding up the volume of each voxel within that cluster.

| Sample | Subjects | Cluster | Volume (ml) | SNP | t | p-value | 95% Confidence Interval | Variance Explained (%) |
|---|---|---|---|---|---|---|---|---|
| Baseline | 1 721 | Left CEN | 1.28±0.17 | rs7182018 | 9.86 | $1.25 \times 10^{-22}$ | (7.93, 11.80) | 5.39 |
| | | Right CEN | 0.83±0.13 | rs7182018 | 9.96 | $4.54 \times 10^{-23}$ | (7.74, 12.18) | 5.50 |
| Follow-up | 869 | Left CEN | 1.23±0.16 | rs7182018 | 6.62 | $3.24 \times 10^{-11}$ | (4,64, 8,59) | 4.87 |
| | | Right CEN | 0.80±0.10 | rs7182018 | 7.31 | $3.04 \times 10^{-13}$ | (5.25, 9.46) | 5.89 |
| SYS | 971 | Left CEN | 1.42±0.30 | rs7182018 | 3.93 | $4.52 \times 10^{-5}$ | (1.91, 5.91) | 1.58 |
| | | Right CEN | 0.86±0.21 | rs7182018 | 4.25 | $1.15 \times 10^{-5}$ | (2.24, 6.51) | 1.84 |
| LIBD HC | 272 | Left CEN | 1.23±0.17 | rs7182018 | 2.88 | $2.15 \times 10^{-3}$ | (1.07, 4.82) | 3.04 |
| | | Right CEN | 0.81±0.11 | rs7182018 | 3.67 | $1.48 \times 10^{-4}$ | (1.60, 5.74) | 4.83 |
| 3C | 515 | Left CEN | 0.64±0.10 | rs7182018 | 4.32 | $9.28 \times 10^{-6}$ | (2.28, 6.38) | 3.55 |
| | | Right CEN | 0.44±0.07 | rs7182018 | 3.84 | $7.02 \times 10^{-5}$ | (1.90, 5.64) | 2.82 |
| LIBD SZ | 157 | Left CEN | 1.14±0.19 | rs7182018 | 2.61 | $4.92 \times 10^{-3}$ | (0.93, 4.48) | 4.35 |
| | | Right CEN | 0.73±0.12 | rs7182018 | 3.22 | $7.78 \times 10^{-4}$ | (1.11, 5.20) | 6.48 |
| LIBD SB | 149 | Left CEN | 1.21±0.18 | rs7182018 | 3.00 | $1.61 \times 10^{-3}$ | (1.14, 5.27) | 5.95 |
| | | Right CEN | 0.78±0.12 | rs7182018 | 1.96 | 0.03 | (0.41, 3.83) | 2.63 |
| UKB | 6 932 | Left CEN | 0.99±0.17 | rs7182018 | 14.83 | $2.19 \times 10^{-49}$ | (12.62,16.88) | 3.09 |
| | | Right CEN | 0.63±0.10 | rs7182018 | 16.35 | $2.87 \times 10^{-59}$ | (14.24,18.39) | 3.73 |

**eTable 6. GWAS Results in Different Sexes.**

"F" for female, "M" for male. P values were given by testing if beta is greater than 0. The mean volumes of each cluster had been compared between female and male by one-way analysis of variance, and the significantly larger volume was marked by "*" for $p < 0.01$, "**" for $p < 0.001$, and "***" for $p < 0.0001$.

| Sample | Subject (F/M) | Cluster | Volumes (ml) | | SNP | T | | p-value | |
|---|---|---|---|---|---|---|---|---|---|
| | | | F | M | | F | M | F | M |
| Baseline | 873/848 | Left PUT | 1.91±0.34 | 1.96±0.36* | rs13107325 | 6.31 | 5.67 | $2.24\times10^{-10}$ | $9.73\times10^{-9}$ |
| | | Right PUT | 0.74±0.08 | 0.75±0.10 | rs13107325 | 6.69 | 5.77 | $2.04\times10^{-11}$ | $5.52\times10^{-9}$ |
| | | Left CEN | 1.28±0.17 | 1.27±0.18 | rs7182018 | 7.46 | 6.43 | $1.06\times10^{-13}$ | $1.10\times10^{-10}$ |
| | | Right CEN | 0.84±0.12*** | 0.82±0.13 | rs7182018 | 7.94 | 6.27 | $3.16\times10^{-15}$ | $2.98\times10^{-10}$ |
| Follow- up | 445/424 | Left PUT | 1.75±0.29 | 1.80±0.32* | rs13107325 | 2.86 | 5.06 | $2.25\times10^{-3}$ | $3.23\times10^{-7}$ |
| | | Right PUT | 0.69±0.08 | 0.70±0.09 | rs13107325 | 2.94 | 5.65 | $1.73\times10^{-3}$ | $1.54\times10^{-8}$ |
| | | Left CEN | 1.25±0.15** | 1.21±0.16 | rs7182018 | 4.86 | 4.43 | $8.28\times10^{-7}$ | $6.01\times10^{-6}$ |
| | | Right CEN | 0.82±0.10*** | 0.78±0.10 | rs7182018 | 4.07 | 6.06 | $2.75\times10^{-5}$ | $1.51\times10^{-9}$ |
| SYS | 504/467 | Left PUT | 1.52±0.20 | 1.69±0.22*** | rs13107325 | 2.55 | 2.64 | $5.47\times10^{-3}$ | $4.30\times10^{-3}$ |
| | | Right PUT | 0.77±0.10 | 0.86±0.11*** | rs13107325 | -1.28 | -1.20 | 0.10 | 0.12 |
| | | Left CEN | 1.39±0.27 | 1.46±0.21** | rs7182018 | 3.31 | 2.12 | $4.99\times10^{-4}$ | 0.02 |
| | | Right CEN | 0.86±0.22 | 0.87±0.22 | rs7182018 | 4.26 | 1.52 | $1.24\times10^{-5}$ | 0.06 |
| LIBD HC | 156/116 | Left PUT | 1.60±0.23 | 1.56±0.21 | rs13107325 | 3.61 | 3.24 | $2.07\times10^{-4}$ | $7.97\times10^{-4}$ |
| | | Right PUT | 0.65±0.06 | 0.64±0.06 | rs13107325 | 4.07 | 3.12 | $3.84\times10^{-5}$ | $1.14\times10^{-3}$ |
| | | Left CEN | 1.26±0.18* | 1.18±0.15 | rs7182018 | 3.14 | 0.67 | $1.00\times10^{-3}$ | 0.25 |
| | | Right CEN | 0.83±0.11 | 0.78±0.11 | rs7182018 | 2.70 | 2.28 | $3.87\times10^{-3}$ | 0.01 |
| 3C | 293/222 | Left PUT | 1.08±0.13 | 1.16±0.15*** | rs13107325 | 0.92 | 2.57 | 0.18 | $5.2\times10^{-3}$ |
| | | Right PUT | 0.46±0.06 | 0.50±0.06*** | rs13107325 | 1.40 | 1.95 | 0.08 | 0.03 |
| | | Left CEN | 0.64±0.09 | 0.64±0.11 | rs7182018 | 2.89 | 3.16 | $2\times10^{-3}$ | $8.48\times10^{-4}$ |

| | | | | | | | | | |
|---|---|---|---|---|---|---|---|---|---|
| | | Right CEN | 0.44±0.06 | 0.44±0.07 | rs7182018 | 2.77 | 2.66 | $2.9\times10^{-3}$ | $4\times10^{-3}$ |
| UKB | 3584/3348 | Left PUT | 1.36±0.23 | 1.39±0.18** | rs13107325 | 2.80 | 4.01 | $2.5\times10^{-3}$ | $3.09\times10^{-5}$ |
| | | Right PUT | 0.52±0.07 | 0.54±0.10*** | rs13107325 | 4.68 | 4.65 | $1.48\times10^{-6}$ | $1.74\times10^{-6}$ |
| | | Left CEN | 0.95±0.14 | 1.03±0.32*** | rs7182018 | 11.85 | 9.53 | $4.25\times10^{-32}$ | $1.49\times10^{-21}$ |
| | | Right CEN | 0.60±0.09 | 0.65±0.10*** | rs7182018 | 11.54 | 10.86 | $2.44\times10^{-35}$ | $2.60\times10^{-27}$ |
| LIBD SZ | 35/122 | Left PUT | 1.68±0.28 | 1.54±0.27 | rs13107325 | 2.22 | 1.59 | 0.02 | 0.06 |
| | | Right PUT | 0.68±0.09 | 0.64±0.08 | rs13107325 | 1.63 | -0.04 | 0.06 | 0.48 |
| | | Left CEN | 1.18±0.21 | 1.13±0.18 | rs7182018 | 1.16 | 2.37 | 0.13 | $9.67\times10^{-3}$ |
| | | Right CEN | 0.78±0.12** | 0.71±0.12 | rs7182018 | 1.12 | 3.22 | 0.14 | $8.23\times10^{-4}$ |
| LIBD SB | 85/64 | Left PUT | 1.55±0.20 | 1.50±0.21 | rs13107325 | 1.92 | 1.24 | 0.03 | 0.11 |
| | | Right PUT | 0.64±0.05 | 0.61±0.07 | rs13107325 | 1.26 | 0.68 | 0.11 | 0.25 |
| | | Left CEN | 1.23±0.19 | 1.18±0.16 | rs7182018 | 3.39 | 0.24 | $5.50\times10^{-4}$ | 0.41 |
| | | Right CEN | 0.81±0.12* | 0.74±0.12 | rs7182018 | 2.06 | 0.49 | 0.02 | 0.31 |

**eTable 7. Association results given by different thresholds using the IMAGEN sample.**

| Threshold | Cluster | Volume (ml) | SNP | $T_{1705}$ | p-value | 95% Confidence Interval | Variance Explained (%) |
|---|---|---|---|---|---|---|---|
| $1.1412\times10^{-13}$ | Left PUT | 1.83±0.33 | rs13107325 | 8.45 | $3.01\times10^{-17}$ | (6.39,10.55) | 4.02 |
| | Right PUT | 0.64±0.08 | rs13107325 | 8.79 | $1.76\times10^{-18}$ | (6.63,11.12) | 4.33 |
| | Left CEN | 1.21±0.16 | rs7182018 | 9.85 | $1.30\times10^{-22}$ | (7.85,11.95) | 5.38 |
| | Right CEN | 0.79±0.12 | rs7182018 | 9.89 | $9.37\times10^{-23}$ | (7.75,12.21) | 5.42 |
| $1.9417\times10^{-11}$ | Left PUT | 2.60±0.43 | rs13107325 | 8.42 | $4.10\times10^{-17}$ | (6.34,10.65) | 3.98 |
| | Right PUT | 1.51±0.18 | rs13107325 | 8.47 | $2.53\times10^{-17}$ | (6.41,10.71) | 4.04 |
| | Left CEN | 1.77±0.22 | rs7182018 | 9.79 | $2.39\times10^{-22}$ | (7.92,11.91) | 5.31 |
| | Right CEN | 1.16±0.17 | rs7182018 | 9.66 | $7.84\times10^{-22}$ | (7.55,11.82) | 5.18 |

**eTable 8. Association results given by different thresholds using the UKB sample.**

| Threshold | Cluster | Volume (ml) | SNP | $T_{6885}$ | p-value | 95% Confidence Interval | Variance Explained (%) |
|---|---|---|---|---|---|---|---|
| $1.1412\times10^{-13}$ | Left PUT | 1.30±0.26 | rs13107325 | 4.77 | $9.52\times10^{-7}$ | (2.94,6.68) | 0.33 |
| | Right PUT | 0.46±0.07 | rs13107325 | 6.52 | $3.71\times10^{-11}$ | (4.52,8.47) | 0.61 |
| | Left CEN | 0.94±0.16 | rs7182018 | 14.9 | $9.00\times10^{-50}$ | (12.68,16.96) | 3.13 |
| | Right CEN | 0.59±0.10 | rs7182018 | 16.34 | $2.90\times10^{-59}$ | (14.24,18.38) | 3.74 |
| $1.9417\times10^{-11}$ | Left PUT | 1.85±0.34 | rs13107325 | 4.91 | $4.64\times10^{-7}$ | (3.07,6.84) | 0.34 |
| | Right PUT | 1.07±0.17 | rs13107325 | 6.10 | $5.70\times10^{-10}$ | (4.16,8.04) | 0.54 |
| | Left CEN | 1.35±0.22 | rs7182018 | 14.36 | $2.16\times10^{-46}$ | (12.13,16.38) | 2.91 |
| | Right CEN | 0.87±0.14 | rs7182018 | 16.23 | $1.88\times10^{-58}$ | (14.10,18.30) | 3.68 |

**eTable 9. Inputs for meta-analysis using the R package metafor.**

The associations between brain clusters and SNPs were estimated by linear regression model as described in the eMethods of vGWAS. Both estimates of the beta coefficient and its standard error were listed. Left PUT stands for the association between SNP rs13107325 and grey matter volume of the cluster in the left putamen. Right PUT stands for the association between rs13107325 and the right putamen cluster; Left CEN is for the association between rs7182018 and the cluster in the left central sulcus; Right CEN is for the association between rs7182018 and the cluster in the right central sulcus

| | Beta | | | | Standard Error | | | |
|---|---|---|---|---|---|---|---|---|
| | Left PUT | Right PUT | Left CEN | Right CEN | Left PUT | Right PUT | Left CEN | Right CEN |
| IMAGEN_BL | 0.1613 | 0.044 | 0.0966 | 0.0648 | 0.0191 | 0.005 | 0.0098 | 0.0066 |
| SYS | 0.0662 | -0.0159 | 0.0809 | 0.0648 | 0.0179 | 0.0092 | 0.0206 | 0.0152 |
| LIBD_HC | 0.1558 | 0.0461 | 0.0783 | 0.0646 | 0.0316 | 0.0086 | 0.0272 | 0.0176 |
| UKB | 0.0388 | 0.0167 | 0.058 | 0.0387 | 0.0081 | 0.0026 | 0.0039 | 0.0024 |
| 3C | 0.0393 | 0.0244 | 0.0389 | 0.0171 | 0.0091 | 0.0064 | 0.0166 | 0.0075 |

**eTable 10. Results of meta-analysis of the SNP— volume associations.**
Meta-analyses were conducted using the R package *metafor* with the inputs listed in eTable 9. The discovery sample (IMAGEN baseline) and the replication samples (SYS, LIBD healthy control, UKB, and 3C) were included in this meta-analysis. The meta estimate of the beta, its standard error (SE), and its 95% confidence interval (ci.lb is the lower bound of the CI, while ci.ub is its upper bound) were listed. The corresponding Z statistic and the p-value were also reported.

| | Estimate | SE | Z | p | ci.lb | ci.ub |
|---|---|---|---|---|---|---|
| **Left PUT** | 0.0547 | 0.0054 | 10.12 | $4.57\times10^{-24}$ | 0.0441 | 0.0653 |
| **Right PUT** | 0.0221 | 0.0021 | 10.7925 | $3.74\times10^{-27}$ | 0.0181 | 0.0262 |
| **Left CEN** | 0.063 | 0.0035 | 18.1922 | $5.95\times10^{-74}$ | 0.0562 | 0.0697 |
| **Right CEN** | 0.0406 | 0.0021 | 19.1043 | $2.32\times10^{-81}$ | 0.0364 | 0.0447 |

**eTable 11. A SNP to whole-brain replication using the UK Biobank sample.**
Given the large sample size of the UK Biobank cohort, we further replicated our findings by the following two whole-brain approaches: 1) testing if the peak voxels within the significant clusters could reach the significance level of 1.9417e-11; 2) testing if the pre-defined clusters were significant by randomized permutation test at a cluster level. We first calculated the mean T ($T_0$) statistic of the associations between the SNP and all voxels within the mask of the significant cluster. Second, we randomly permuted the genotype data 10,000 times, calculated its association with each voxel in the brain, identified the peak voxel with the strongest association, selected the top N voxels (N was the number of voxels in the corresponding cluster defined by the mask of the discovery results listed in eTable 2) within its neighbourhood with the strongest associations, and then estimated the mean T statistic of the associations ($T_{perm}$). Finally, we added one to the count (S) if $T_{perm} > T_0$ at each permutation, and the significance level of this randomized permutation test was given by $p_{perm} = S/10,000$.

| Marker | A1 | A2 | MAF | Name | Cluster size (number of voxels) | Peak within cluster | | | | | p-value given by Cluster-level permutation |
|---|---|---|---|---|---|---|---|---|---|---|---|
| | | | | | | X | Y | Z | $t_{6885}$ | p | |
| rs13107325 | C | T | 0.07 | L.PUT | 639 | -13.5 | 7.5 | -9 | 6.73 | $1.80\times10^{-11}$ | 0.0001 |
| | | | | R.PUT | 263 | 31.5 | -9 | -3 | 8.60 | $9.49\times10^{-18}$ | <0.0001 |
| rs7182018 | A | G | 0.09 | L.CEN | 710 | -52.5 | -6 | 19.5 | 16.52 | $4.02\times10^{-60}$ | <0.0001 |
| | | | | R.CEN | 452 | 55.5 | -3 | 19.5 | 17.94 | $2.09\times10^{-70}$ | <0.0001 |

**eTable 12. Gene expression associations of rs7182018.**
We queried the SNP **rs7182018** (chr15:39632269) at the website http://www.braineac.org/ and it returned the following significant association results. Those p-values survived the threshold 0.05/10 (brain tissues) / 2 (nearby genes) = 0.0025 were marked in bold.

| Gene Symbol | exprID | aveALL | CRBL | FCTX | HIPP | MEDU | OCTX | PUTM | SNIG | TCTX | THAL | WHMT |
|---|---|---|---|---|---|---|---|---|---|---|---|---|
| THBS1 | 3589485 | 2.1E-02 | 7.7E-01 | **1.4E-04** | 4.7E-01 | 7.9E-01 | 9.0E-01 | 6.8E-01 | 2.3E-01 | 1.3E-02 | 1.3E-01 | 2.3E-01 |
| C15orf52 | 3619413 | 1.8E-01 | 4.7E-01 | **2.3E-04** | 9.0E-01 | 9.9E-01 | 2.7E-01 | 4.1E-01 | 3.8E-02 | 7.0E-01 | 7.6E-01 | 2.1E-01 |
| C15orf53 | t3589290 | 2.7E-03 | 8.6E-01 | 5.9E-02 | 5.0E-02 | **6.7E-04** | 2.6E-01 | 2.4E-01 | 3.7E-01 | 3.5E-01 | 9.4E-01 | 5.3E-01 |
| C15orf52 | 3619425 | **1.6E-03** | 3.4E-01 | 1.4E-02 | 2.7E-01 | 3.4E-01 | 5.7E-01 | 7.3E-01 | 1.1E-01 | 2.8E-02 | 2.0E-02 | 7.9E-01 |
| C15orf56 | t3619310 | 2.2E-01 | 5.4E-01 | **2.1E-03** | 7.6E-02 | 7.3E-01 | 8.2E-01 | 8.3E-01 | 7.8E-01 | 3.5E-02 | 7.1E-01 | 2.0E-01 |

**eTable 13. Gene expression associations of rs13107325.**

We queried the SNP rs13107325 (chr4: 103188709) at the website http://www.braineac.org/ and it returned the following significant association results. Those p-values survived the threshold 0.05/10/6 = 8.33e-04 were marked in bold.

| Gene Symbol | exprID | aveALL | CRBL | FCTX | HIPP | MEDU | OCTX | PUTM | SNIG | TCTX | THAL | WHMT |
|---|---|---|---|---|---|---|---|---|---|---|---|---|
| SLC39A8 | 2779840 | 6.2e-01 | 3.8e-02 | 9.8e-01 | 5.1e-01 | 4.9e-01 | 1.3e-01 | **1.7e-04** | 3.5e-01 | 9.9e-01 | 5.7e-01 | 9.8e-01 |
| SLC39A8 | 2779842 | 7.4e-01 | 3.6e-02 | 7.8e-01 | 1.1e-01 | 3.4e-01 | 8.6e-02 | **2.0e-04** | 6.4e-01 | 7.3e-01 | 2.6e-01 | 4.9e-01 |
| MANBA | 2779900 | 1.2e-01 | 4.3e-01 | **2.9e-04** | 8.9e-01 | 6.7e-01 | 4.9e-01 | 9.5e-01 | 3.6e-01 | 5.8e-01 | 1.7e-01 | 2.2e-01 |
| CENPE | 2780247 | 4.2e-01 | 4.7e-01 | 8.6e-01 | 8.5e-01 | 7.2e-01 | **3.3e-04** | 7.0e-01 | 1.9e-01 | 7.5e-01 | 6.3e-02 | 6.0e-01 |
| NFKB1 | 2737753 | 5.5e-01 | 5.8e-01 | 6.2e-01 | **4.3e-04** | 3.6e-01 | 6.3e-01 | 1.7e-02 | 5.0e-01 | 3.3e-01 | 3.6e-01 | 9.1e-01 |
| SLC39A8 | 2779831 | 5.1e-02 | 5.6e-01 | 7.8e-01 | 4.0e-01 | 3.1e-01 | 2.8e-01 | **7.5e-04** | 8.7e-01 | 9.4e-01 | 5.7e-01 | 6.1e-01 |
| SLC39A8 | 2779827 | 4.9e-01 | 1.6e-02 | 8.9e-01 | 5.3e-01 | 6.7e-01 | 4.1e-01 | 1.2e-03 | 8.4e-01 | 7.7e-01 | 6.5e-01 | 8.9e-01 |
| SLC39A8 | 2779843 | 6.0e-01 | 5.0e-02 | 9.3e-01 | 5.3e-01 | 8.2e-01 | 4.6e-01 | 1.2e-03 | 8.4e-01 | 8.5e-01 | 3.2e-01 | 7.9e-01 |
| CENPE | 2780194 | 5.1e-01 | 4.1e-01 | 5.6e-01 | 1.3e-03 | 8.6e-01 | 9.0e-01 | 6.5e-01 | 9.0e-03 | 1.7e-01 | 6.6e-01 | 2.4e-01 |
| SLC39A8 | 2779826 | 1.4e-02 | 3.6e-02 | 8.4e-02 | 5.5e-01 | 8.6e-01 | 1.5e-03 | 9.7e-03 | 2.6e-01 | 4.6e-01 | 7.5e-01 | 4.3e-01 |

**eTable 14. LD matrices D’ (R2) for significant SNP’s.**

We estimated D’ and $R^2$ for the significant SNP’s identified in eFigure 17 by https://ldlink.nci.nih.gov/?tab= ldmatrix for 1000 Genome Project CEU population.

| **RS_ID** | **rs7182018** | **rs11638679** | |
|---|---|---|---|
| **rs8025239** | 0.693(0.452) | 0.776(0.173) | |
| **rs7182018** | | 1(0.306) | |
| | | | |
| **RS_ID** | **rs13107325** | **rs13114738** | **rs13116385** |
| **rs17199964** | 0.745(0.367) | 0.391(0.082) | 0.475(0.115) |
| **rs13107325** | | 0.762(0.469) | 0.82(0.518) |
| **rs13114738** | | | 0.951(0.861) |

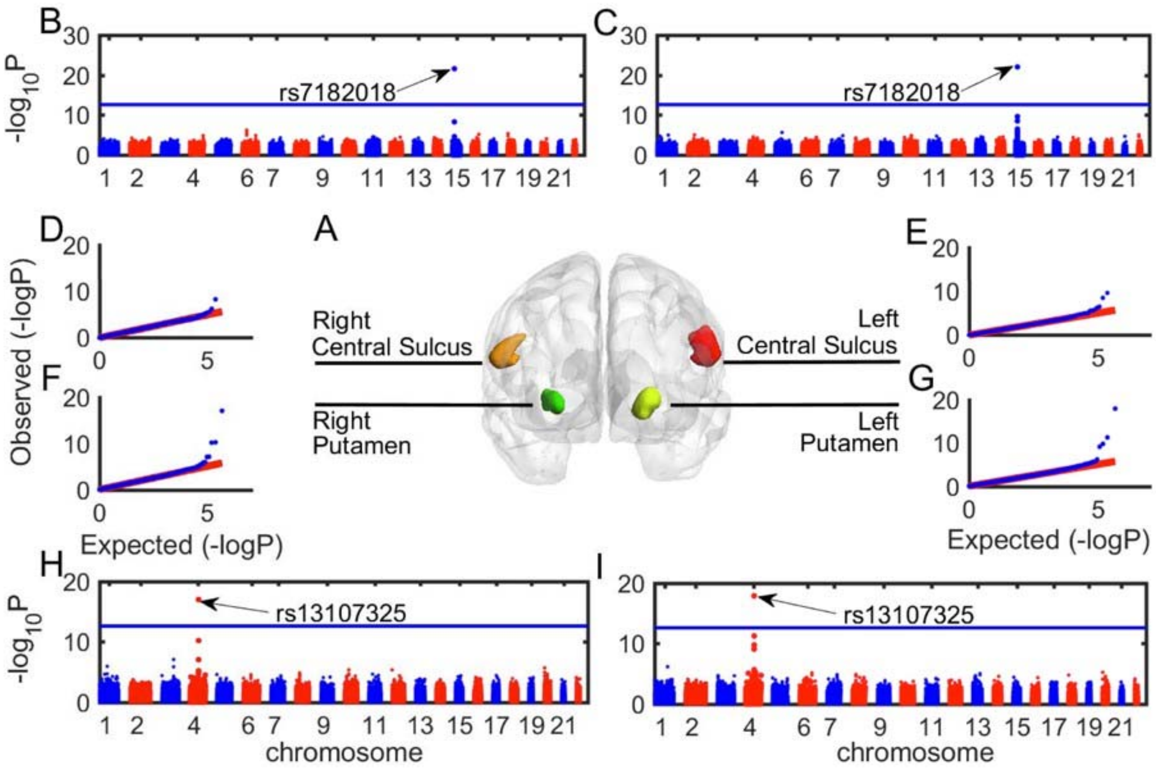


**eFigure 1. GWAS on four clusters reached the brain-wide and genome-wide significance level.**

Common genetic variants associated with two clusters in the bilateral putamen and two clusters around the bilateral central sulcus. Genome wide significance level was $p < 2.4483\times10^{-13} = 0.05/438145/466114$ corrected for number of voxels and number of genetic variants (blue line). A) The 3D image shows the definition of these four ROI's. The genome wide association results, including Manhattan plot (B, C, H, I) and QQ plot (D, E, F, G) were shown for four ROI's, left central sulcus, right central sulcus, right putamen, and left putamen, respectively.

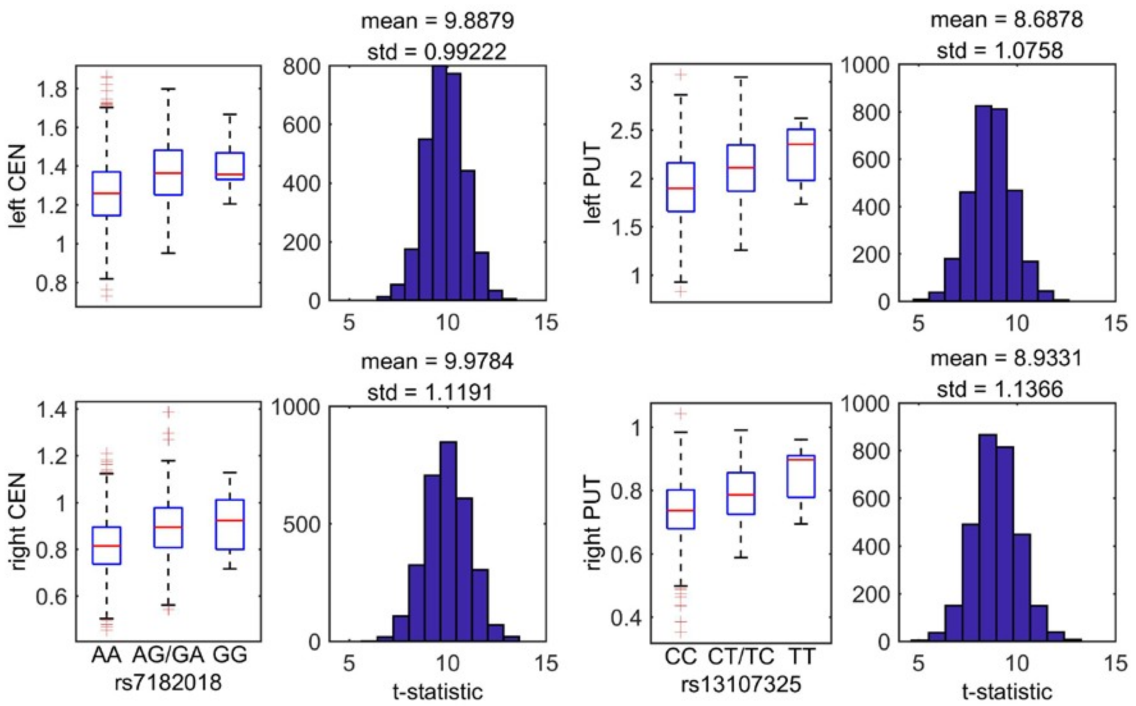


**eFigure 2. Comparison of volumes and histogram of t-statistics in IMAGEN baseline sample.**
Boxplots of grey matter volumes grouped by genotype and histogram of t-statistic by 3000 bootstraps in the IMAGEN sample at the baseline.

A

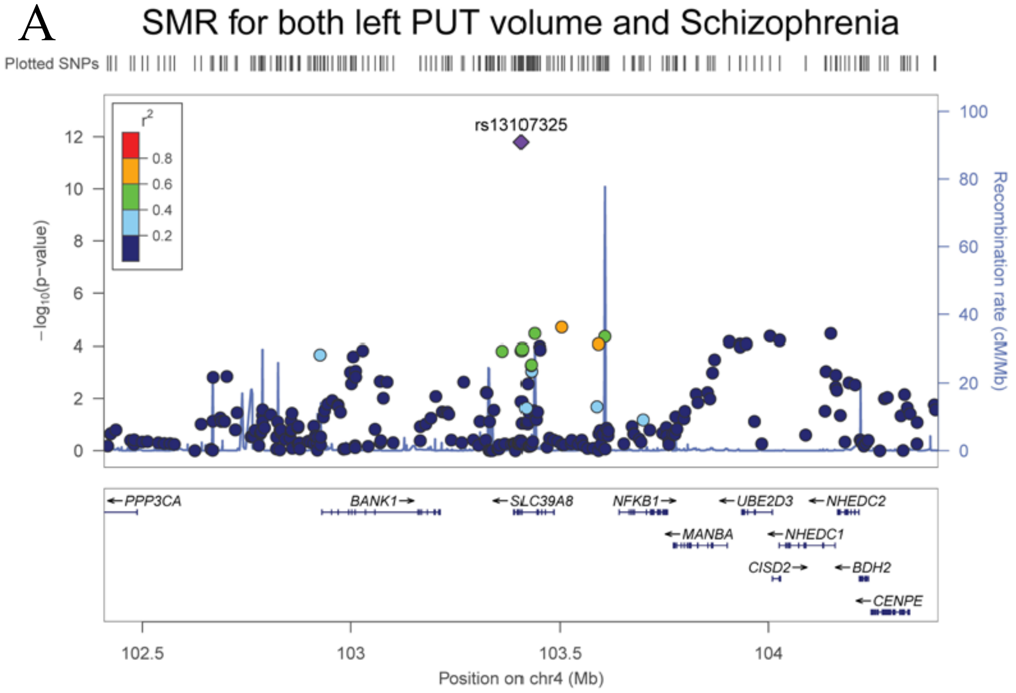


B

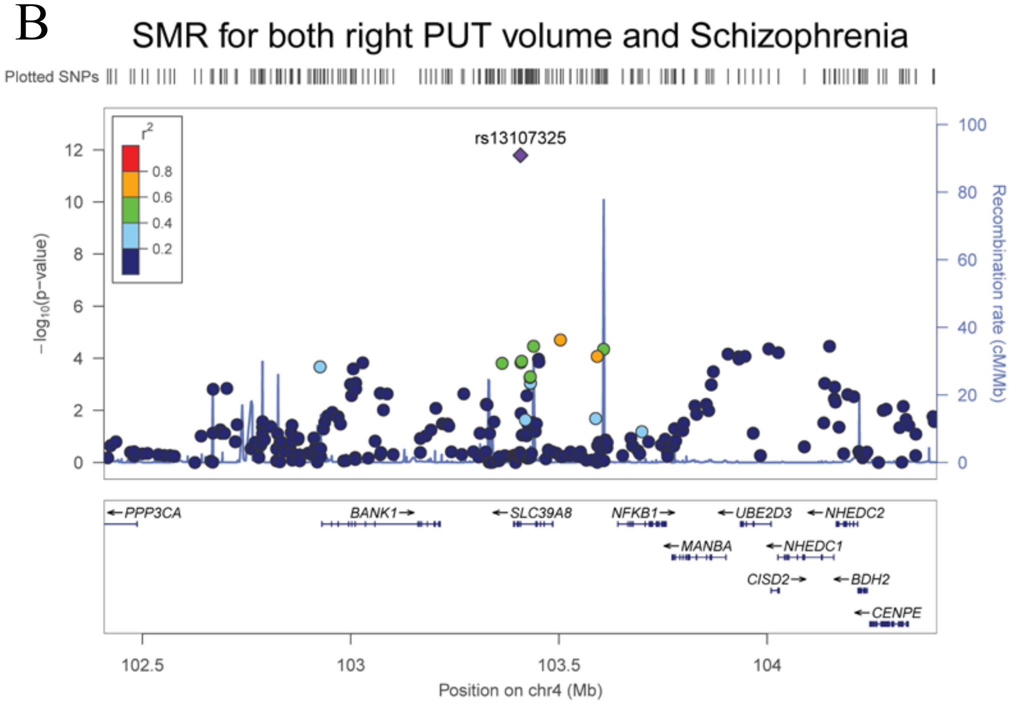


**eFigure 3. The association between putamen volume and schizophrenia free of non-genetic confounders given by SMR analysis using each SNP as an instrument.**
Significance level (p-value) given by Wald ratio test in SMR analysis (putamen volume as an exposure, schizophrenia as an output, and each SNP as an instrument; http://www.mrbase.org) were plotted with recombination rate for ±1M region of the location of rs13107325 on the 4th chromosome. The figure was generated by LocusZoom (http://locuszoom.org). A) for the cluster in the left putamen; B) for the cluster in the right putamen.

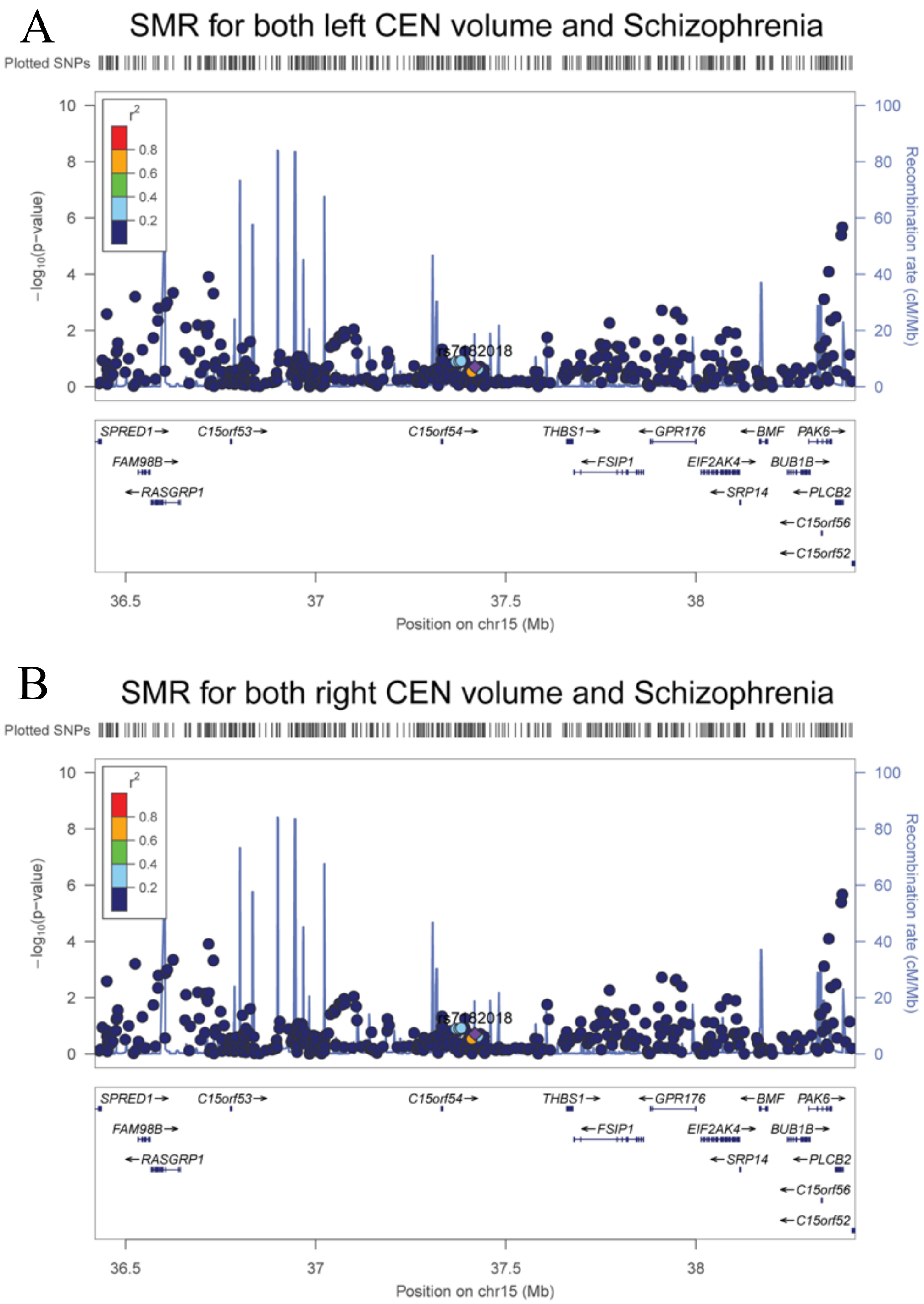


**eFigure 4. The association between central sulcus volume and schizophrenia free of non-genetic confounders given by SMR analysis using each SNP as an instrument.**

Significance level (p-value) given by Wald ratio test in SMR analysis (central sulcus volume as an exposure, schizophrenia as an output, and each SNP as an instrument; http://www.mrbase.org) were plotted with recombination rate for ±1M region of the location of rs7182018 on the 4th chromosome. The figure was generated by LocusZoom (http://locuszoom.org). A) for the cluster in the left central sulcus; B) for the cluster in the right central.

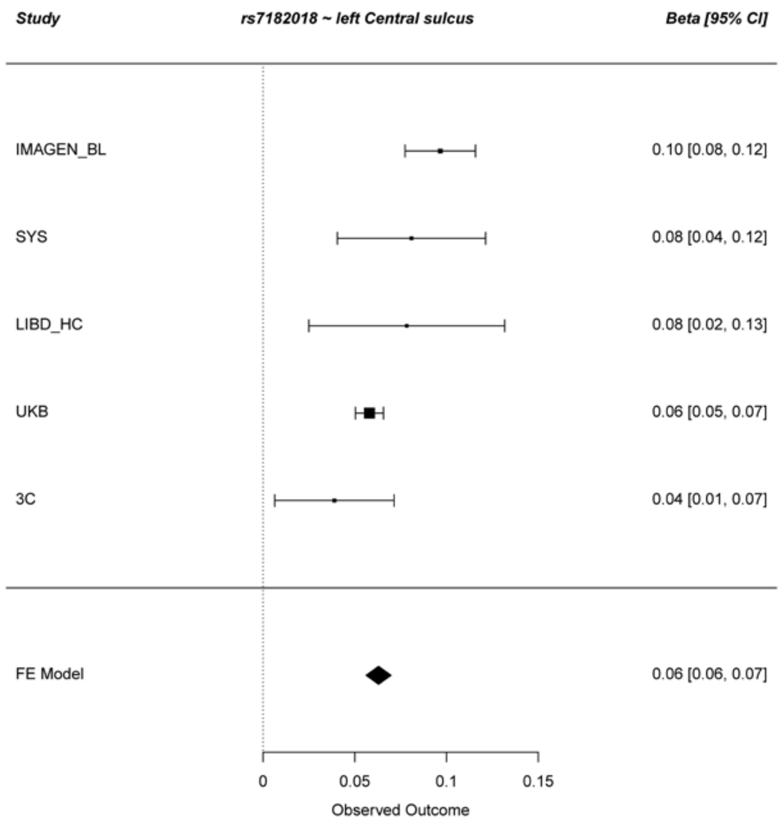


**eFigure 5. Forest plot of the meta-analysis in the left central sulcus.**
The forest plot for the meta-analysis of the association between rs7182018 and the grey matter volume of the cluster in the left central sulcus. This figure was generated by the R package *metafor*.

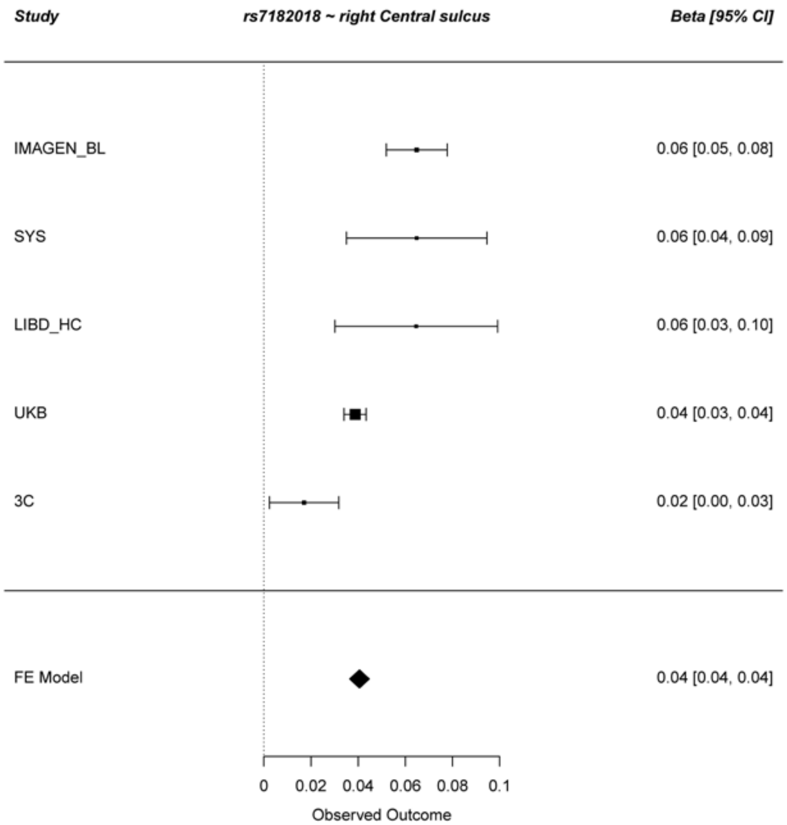


**eFigure 6. Forest plot of the meta-analysis in the right central sulcus.**
The forest plot for the meta-analysis of the association between rs7182018 and the grey matter volume of the cluster in the right central sulcus. This figure was generated by the R package *metafor*.

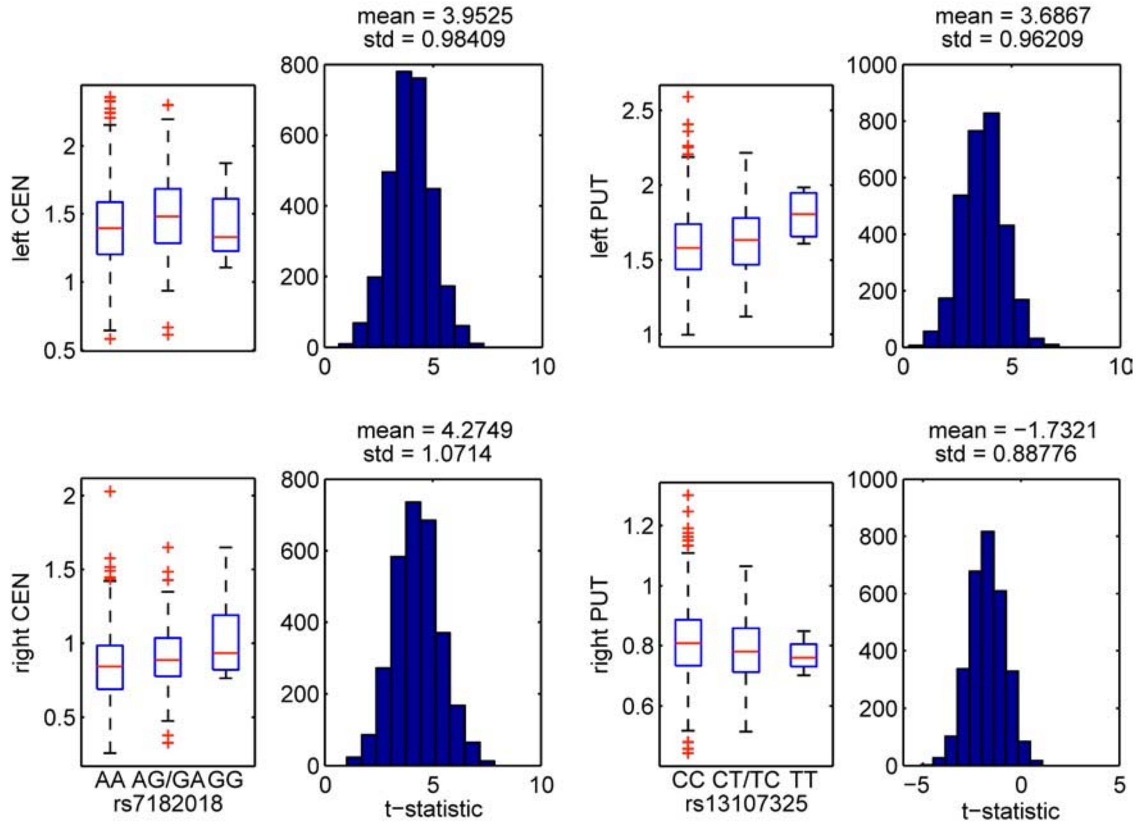


**eFigure 7. Comparison of volumes and histogram of t-statistics in the SYS sample.**
Boxplots of grey matter volumes grouped by genotype and histogram of t-statistic by 3000 bootstraps in the SYS sample.

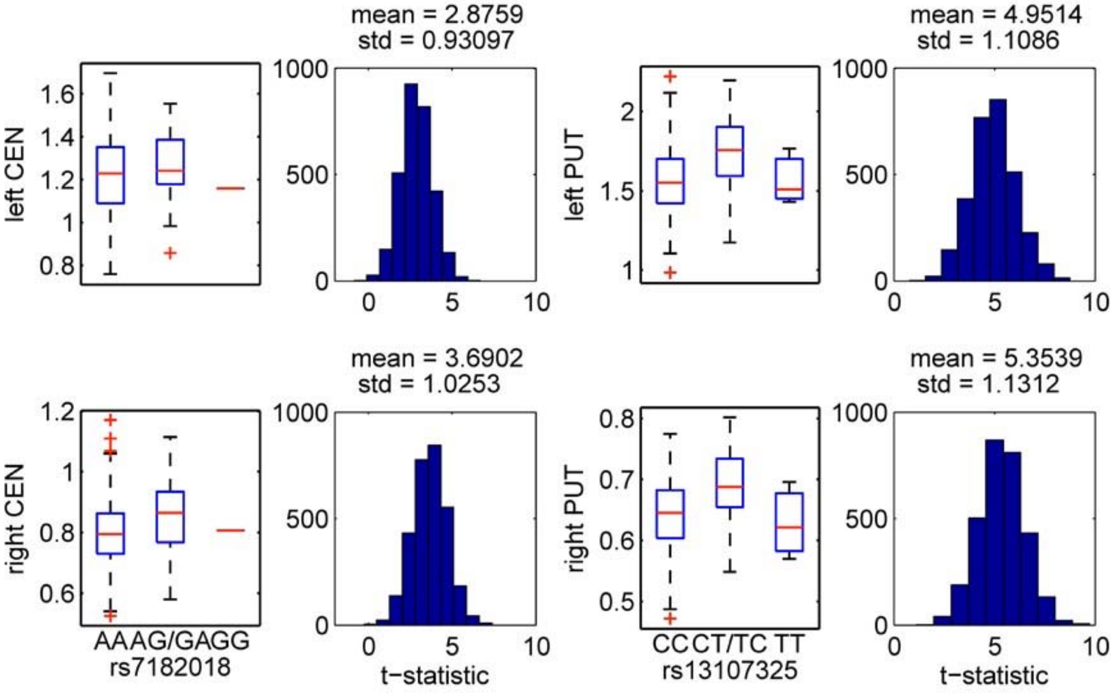


**eFigure 8. Comparison of volumes and histogram of t-statistics in the LIBD healthy controls.**
Boxplots of grey matter volumes grouped by genotype and histogram of t-statistic by 3000 bootstraps in the LIBD healthy controls.

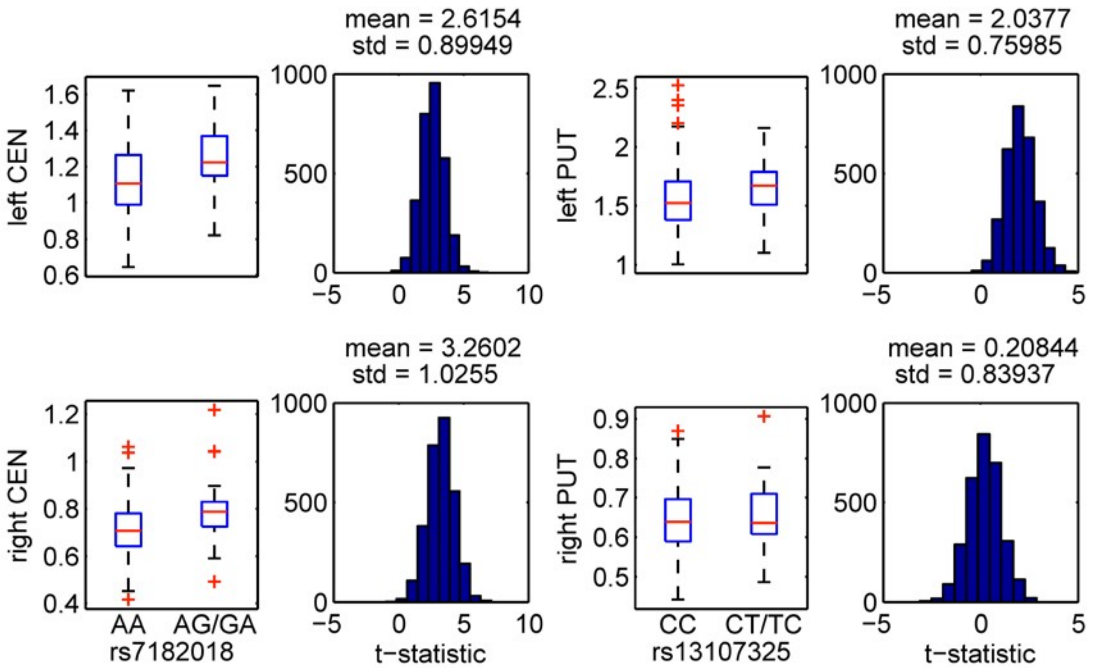


**eFigure 9. Comparison of volumes and histogram of t-statistics in the LIBD patients.**
Boxplots of grey matter volumes grouped by genotype and histogram of t-statistic by 3000 bootstraps for the schizophrenic patients in the LIBD study.

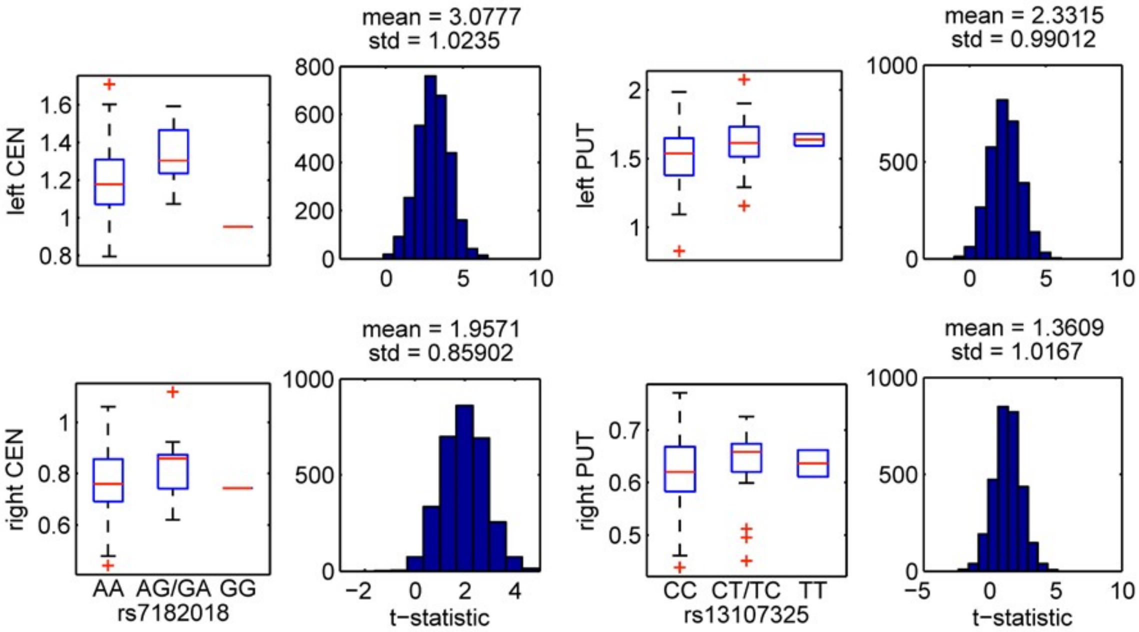


**eFigure 10. Comparison of volumes and histogram of t-statistics for unaffected siblings of patients in the LIBD study.**
Boxplots of grey matter volumes grouped by genotype and histogram of t-statistic by 3000 bootstraps for the unaffected siblings of schizophrenic patients in the LIBD study.

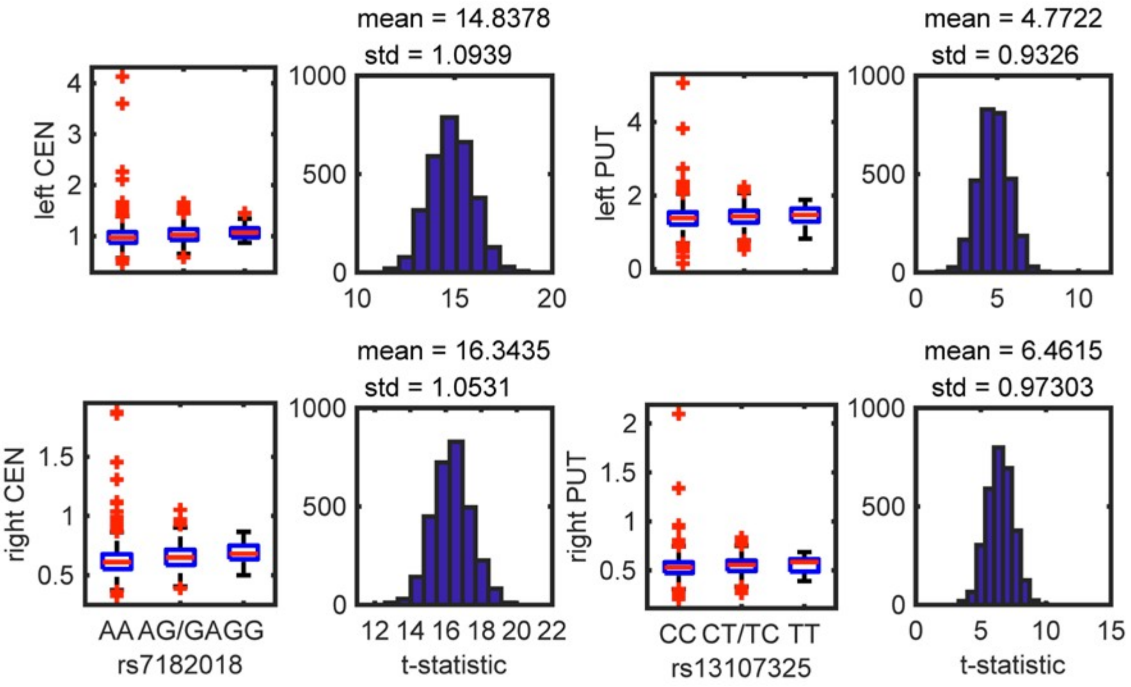


**eFigure 11. Comparison of volumes and histogram of t-statistics in the UKB.**
Boxplots of grey matter volumes grouped by genotype and histogram of t-statistic by 3000 bootstraps in the UKB.

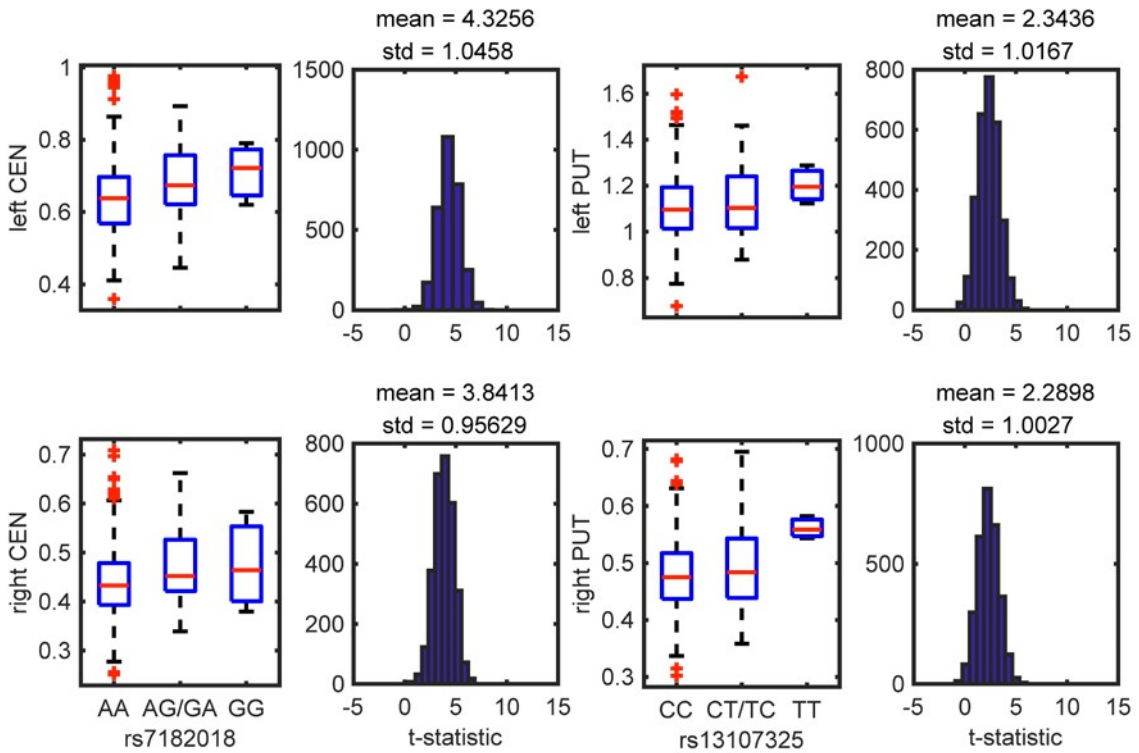


**eFigure 12. Comparison of volumes and histogram of t-statistics in the 3C sample.**
Boxplots of grey matter volumes grouped by genotype and histogram of t-statistic by 3000 bootstraps in the 3C sample.

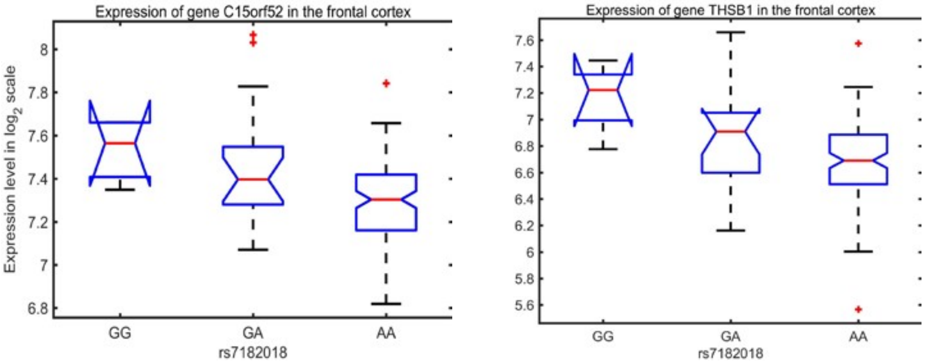


**eFigure 13. Boxplots of gene expression in the frontal cortex.**

We queried these results using the UKBEC database (http://braineac.org).

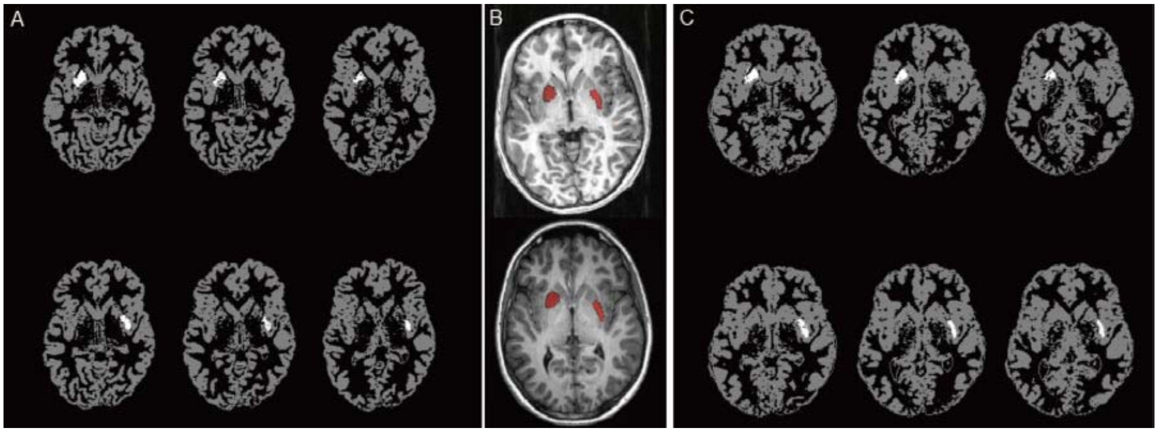


**eFigure 14. Mappings of putamen clusters onto individual space in IMAGEN and SYS.**

A) C) two typical subjects in the SYS sample. B) two typical subjects in the IMAGEN sample. The clusters in putamen were defined by the vGWAS in the IMAGEN sample in the standard MNI space, and then mapped into individual spaces of the SYS sample by the method described in the Method section of the main text. As we can see from these typical examples, the left cluster in the anterior putamen was mapped well in the SYS sample, but the right cluster was contaminated by the insula where the anatomy varies greatly across subjects, and this variation may be much greater than that of putamen. Examples of such variations for other cortical regions (cingulate and paracingulate sulci) are well-established[18].

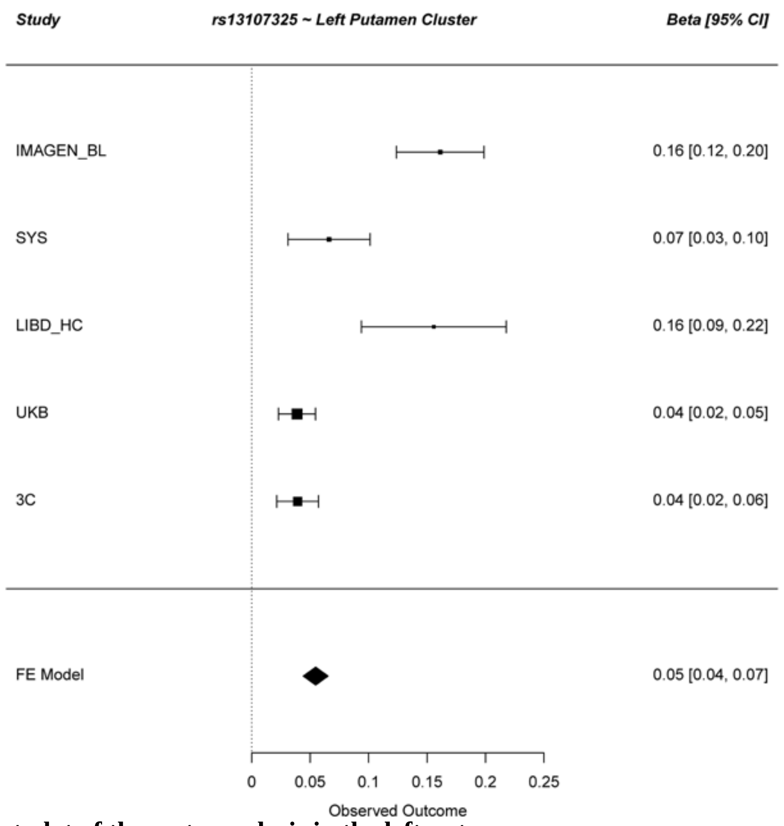


**eFigure 15. Forest plot of the meta-analysis in the left putamen.**

The forest plot for the meta-analysis of the association between rs13107325 and the grey matter volume of the cluster in the left putamen. This figure was generated by the R package *metafor*.

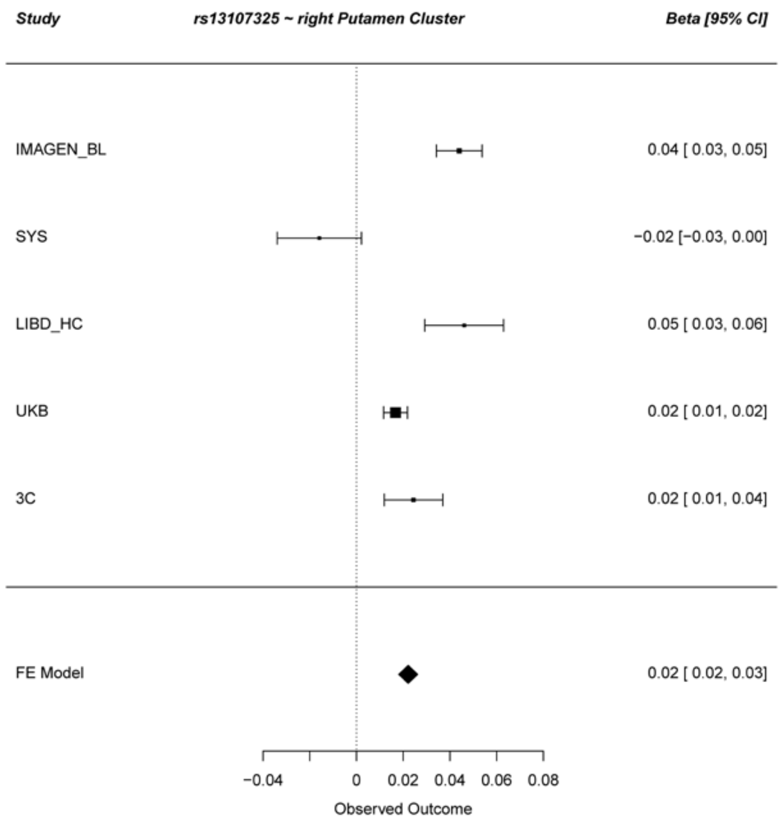


**eFigure 16. Forest plot of the meta-analysis in the right putamen.**

The forest plot for the meta-analysis of the association between rs13107325 and the grey matter volume of the cluster in the right putamen. This figure was generated by the R package *metafor*.

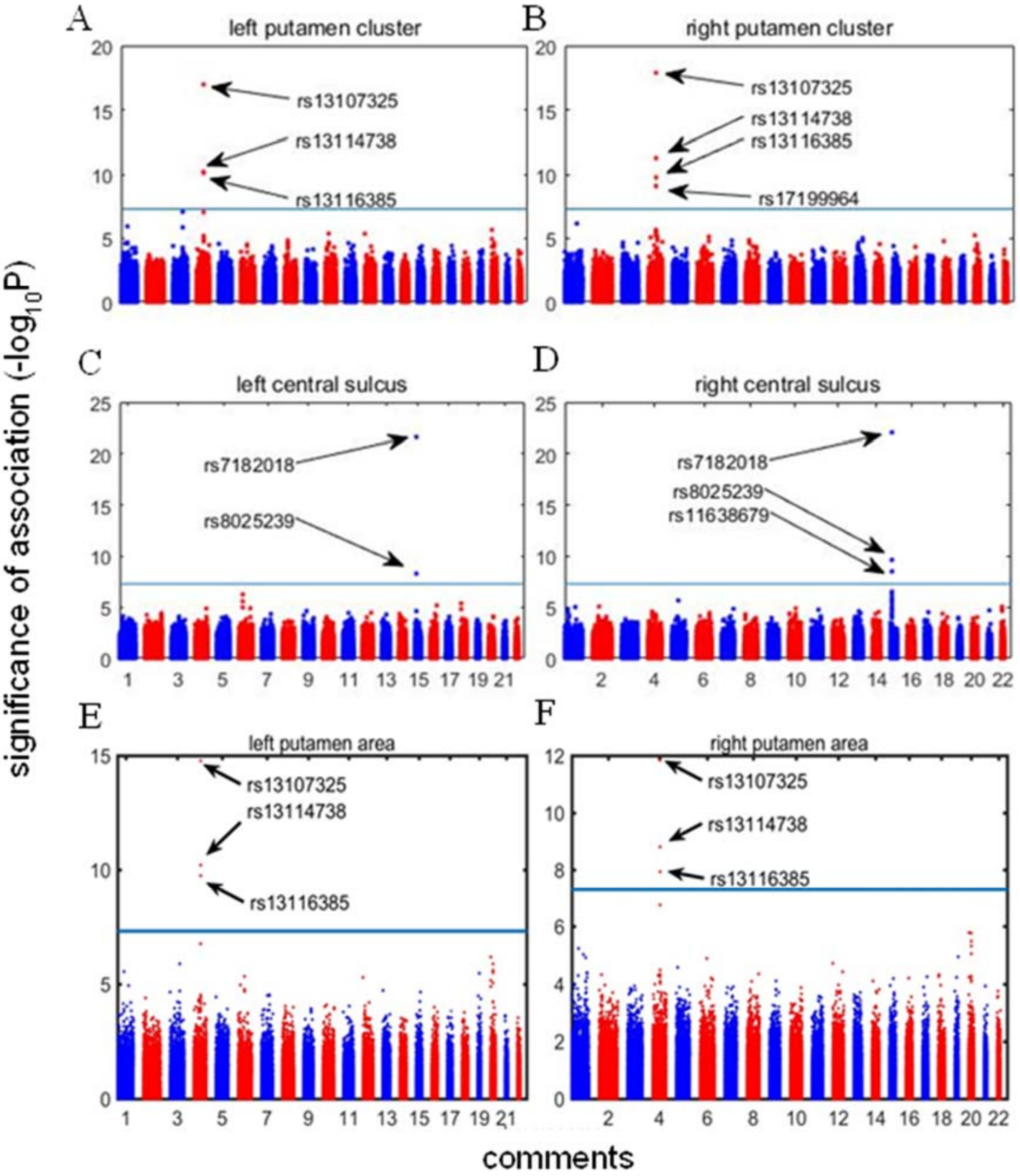


**eFigure 17.** GWAS signals for GMV using the genome-wide significance level (p < 5e-8).

**A**. left putamen cluster; **B**. right putamen cluster; **C**. left central sulcus cluster; **D**. right central sulcus cluster; **E**. left putamen brain area; **F**. right putamen brain area.

**Reference**


1. Price AL, Patterson NJ, Plenge RM, Weinblatt ME, Shadick NA, Reich D. Principal components analysis corrects for stratification in genome-wide association studies. *Nature genetics.* 2006;38(8):904-909.
2. Pausova Z, Paus T, Abrahamowicz M, et al. Cohort Profile: The Saguenay Youth Study (SYS). *International Journal of Epidemiology.* 2016.
3. The Genomes Project C. An integrated map of genetic variation from 1,092 human genomes. *Nature.* 2012;491:56.
4. Delaneau O, Zagury J-F, Marchini J. Improved whole-chromosome phasing for disease and population genetic studies. *Nature Methods.* 2012;10:5.
5. Howie BN, Donnelly P, Marchini J. A Flexible and Accurate Genotype Imputation Method for the Next Generation of Genome-Wide Association Studies. *PLOS Genetics.* 2009;5(6):e1000529.
6. Honea RA, Meyer-Lindenberg A, Hobbs KB, et al. Is Gray Matter Volume an Intermediate Phenotype for Schizophrenia? A VBM Study of Patients with Schizophrenia and their Healthy Siblings. *Biological psychiatry.* 2008;63(5):465-474.
7. Miller KL, Alfaro-Almagro F, Bangerter NK, et al. Multimodal population brain imaging in the UK Biobank prospective epidemiological study. *Nature neuroscience.* 2016;19(11):1523-1536.
8. Trabzuni D, Ryten M, Walker R, et al. Quality control parameters on a large dataset of regionally dissected human control brains for whole genome expression studies. *Journal of neurochemistry.* 2011;119(2):275-282.
9. Tzourio-Mazoyer N, Landeau B, Papathanassiou D, et al. Automated Anatomical Labeling of Activations in SPM Using a Macroscopic Anatomical Parcellation of the MNI MRI Single-Subject Brain. *Neuroimage.* 2002;15(1):273–289.
10. Li J, Ji L. Adjusting multiple testing in multilocus analyses using the eigenvalues of a correlation matrix. *Heredity.* 2005;95:221.
11. Desrivieres S, Lourdusamy A, Tao C, et al. Single nucleotide polymorphism in the neuroplastin locus associates with cortical thickness and intellectual ability in adolescents. *Mol Psychiatry.* 2015;20(2):263-274.
12. Cheng H, Wu H, Fan Y. Optimizing affinity measures for parcellating brain structures based on resting state fMRI data: a validation on medial superior frontal cortex. *Journal of neuroscience methods.* 2014;237:90-102.
13. Hibar DP, Stein JL, Renteria ME, et al. Common genetic variants influence human subcortical brain structures. *Nature.* 2015;520(7546):224-229.
14. Yan C-G, Wang X-D, Zuo X-N, Zang Y-F. DPABI: Data Processing & Analysis for (Resting-State) Brain Imaging. *Neuroinformatics.* 2016;14(3):339-351.
15. Zhu Z, Zhang F, Hu H, et al. Integration of summary data from GWAS and eQTL studies predicts complex trait gene targets. *Nature genetics.* 2016;48:481.
16. Schizophrenia Working Group of the Psychiatric Genomics C. Biological insights from 108 schizophrenia-associated genetic loci. *Nature.* 2014;511:421.
17. Hemani G, Zheng J, Elsworth B, et al. The MR-Base platform supports systematic causal inference across the human phenome. *eLife.* 2018;7:e34408.

18. Paus T, Otaky N, Caramanos Z, et al. In vivo morphometry of the intrasulcal gray matter in the human cingulate, paracingulate, and superior-rostral sulci: hemispheric asymmetries, gender differences and probability maps. *The Journal of comparative neurology*. 1996;376(4):664-673.
